# Deep learning in spatially resolved transcriptomics: a comprehensive technical view

**DOI:** 10.1093/bib/bbae082

**Published:** 2024-03-13

**Authors:** Roxana Zahedi, Reza Ghamsari, Ahmadreza Argha, Callum Macphillamy, Amin Beheshti, Roohallah Alizadehsani, Nigel H Lovell, Mohammad Lotfollahi, Hamid Alinejad-Rokny

**Affiliations:** UNSW BioMedical Machine Learning Lab (BML), The Graduate School of Biomedical Engineering, UNSW Sydney, 2052, NSW, Australia; UNSW BioMedical Machine Learning Lab (BML), The Graduate School of Biomedical Engineering, UNSW Sydney, 2052, NSW, Australia; The Graduate School of Biomedical Engineering, UNSW Sydney, 2052, NSW, Australia; Tyree Institute of Health Engineering (IHealthE), UNSW Sydney, 2052, NSW, Australia; School of Animal and Veterinary Sciences, University of Adelaide, Roseworthy, 5371, Australia; School of Computing, Macquarie University, Sydney, 2109, Australia; Institute for Intelligent Systems Research and Innovation (IISRI), Deakin University, Waurn Ponds, Melbourne, VIC, 3216, Australia; The Graduate School of Biomedical Engineering, UNSW Sydney, 2052, NSW, Australia; Tyree Institute of Health Engineering (IHealthE), UNSW Sydney, 2052, NSW, Australia; Computational Health Center, Helmholtz Munich, Germany; Wellcome Sanger Institute, Cambridge, UK; UNSW BioMedical Machine Learning Lab (BML), The Graduate School of Biomedical Engineering, UNSW Sydney, 2052, NSW, Australia; Tyree Institute of Health Engineering (IHealthE), UNSW Sydney, 2052, NSW, Australia

**Keywords:** Spatially resolved transcriptomics, deep learning, gene expression, histology images, multimodal analysis

## Abstract

Spatially resolved transcriptomics (SRT) is a pioneering method for simultaneously studying morphological contexts and gene expression at single-cell precision. Data emerging from SRT are multifaceted, presenting researchers with intricate gene expression matrices, precise spatial details and comprehensive histology visuals. Such rich and intricate datasets, unfortunately, render many conventional methods like traditional machine learning and statistical models ineffective. The unique challenges posed by the specialized nature of SRT data have led the scientific community to explore more sophisticated analytical avenues. Recent trends indicate an increasing reliance on deep learning algorithms, especially in areas such as spatial clustering, identification of spatially variable genes and data alignment tasks. In this manuscript, we provide a rigorous critique of these advanced deep learning methodologies, probing into their merits, limitations and avenues for further refinement. Our in-depth analysis underscores that while the recent innovations in deep learning tailored for SRT have been promising, there remains a substantial potential for enhancement. A crucial area that demands attention is the development of models that can incorporate intricate biological nuances, such as phylogeny-aware processing or in-depth analysis of minuscule histology image segments. Furthermore, addressing challenges like the elimination of batch effects, perfecting data normalization techniques and countering the overdispersion and zero inflation patterns seen in gene expression is pivotal. To support the broader scientific community in their SRT endeavors, we have meticulously assembled a comprehensive directory of readily accessible SRT databases, hoping to serve as a foundation for future research initiatives.

## INTRODUCTION

Multicellular organisms have diverse tissues composed of specialized cells that constantly divide and perform specific functions [1]. Cell fate and behavior rely on communication with the surrounding environment, and understanding the spatial organization within tissues is crucial for studying tissue function and disease processes, such as autoimmunity and cancer [2]. Single-cell RNA sequencing (scRNA-seq) has revolutionized genomics by capturing gene activity at a high resolution, enabling the study of heterogeneous cell populations in various disciplines [3]. However, scRNA-seq requires tissue dissociation, leading to the loss of cell position, which is important for understanding tissue functionality. Spatially resolved transcriptomics (SRT) provides a solution by capturing gene expression and spatial information simultaneously across tissues [4]. SRT methods can be divided into image-based methods with high spatial resolution but limited gene detection sensitivity; and sequencing-based methods with lower spatial resolution but high-throughput messenger RNA (mRNA) capture [5]. Image-based methods like *in situ* hybridization (ISH) allow gene expression quantification at a sub-cellular level, while sequencing-based methods rely on spatial barcoding and sequencing [6].

**Figure 1:**
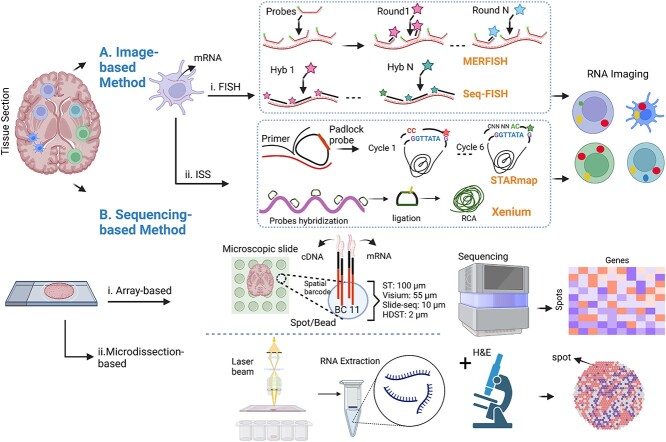
Schematic overview of two SRT approaches. (**A**) **Image-based methods:**  *I. fluorescent in situ hybridization (FISH) approach:* Probes labeled with fluorophores are individually hybridized to predefined RNA targets, allowing visualization of gene expression in fixed tissue. This approach has been enhanced with shorter probes, leading to quantitative measurements of transcripts (smFISH) [23]. Sequential hybridizations (seqFISH) [24] were introduced to expedite the process and multiplexed error-robust FISH (MERFISH) [25] utilized binary codes to distinguish targeted transcript. *II. In situ sequencing methods (ISS):* RNA sequencing is performed directly on the RNA content within the tissue using using padlock probes to target genes. STARmap [26] is an ISS method, employing barcoded padlock probes and additional primers. Xenium [27] is a hybrid ISS and ISH platform that utilizes gene-specific barcoded padlock probes, with the enzymatic amplification step employing the Rolling Circle Amplification technique for enhanced detection sensitivity. (**B**) **Sequencing-based methods:** This category enables unbiased analysis of the complete transcriptome and can be divided into *I.Array and Bead-Based Technologies:* In these methods the targeted tissue is placed on a microscopic slide with a barcoded array, capturing spatial information of each probe. Probes containing spatial barcodes and RT primers are inserted into the tissue. After tissue removal, cDNA-mRNA complexes are extracted for library preparation and next-generation sequencing readout. Gene expressions are measured in spots or beads, accompanied by a high-resolution histology image obtained from stained tissue sections of the same tissue. Probes dimensions vary across technologies, such as 100 $\mu{\mathrm m}$ (ST) or 55 $\mu{\mathrm m}$ (10X Visium), or utilizing ordered bead arrays (HDST) or barcoded beads (Slide-seq) of specific sizes (10 $\mu{\mathrm m}$). *II. Microdissection-Based Approach:* Laser Capture Microdissection (LCM) [28] utilizes a concentrated infrared laser pulse for isolating a chosen region within a tissue sample. This technique ensures the accurate extraction of specimens from targeted anatomical areas, effectively reducing the risk of contamination. It allows for the detailed analysis of transcriptomes at the cellular level [29]. (Created with BioRender.com).

SRT data, along with existing histology images and gene expression data, have generated vast and complex datasets [7] that require statistical and machine learning (ML) methods for analysis. ML methods, particularly deep learning (DL), have proven efficient in various biological tasks and are well suited for handling the challenges of SRT datasets [8–18]. Conventional (ML) methods in the SRT data analysis are mainly similar to the statistical inference domain, in which there is a demand for pre-existing knowledge about the data to estimate unknown parameters in the model [19]. Consequently, the DL method does not need to know the data-generation process to model data and is more potent in extracting complex and high-dimensional features. DL models are more versatile for integrating histology images, gene expression matrices and spatial information. Indeed, DL paradigms have facilitated the handling of such complicated datasets and related downstream analyses. Some efforts have been made to review the computational challenges in the SRT domain. Hu *et al*. [20] focused on the statistical and ML methods to analyze SRT data. This work has focused on leveraging the capabilities of histology images applicable to both imaging-based and sequencing-based techniques. Zeng *et al*. [21] provided a summary of the statistical and ML methods in the SRT domain with more focus on the sequencing-based methods. Despite the valuable information in these review papers, they did not provide detailed information and discussion of the application of DL models in SRT analysis. Although the review paper by Heydari and Sindi [22] reviewed the application of DL in SRT analysis, this work mainly focused on sequencing-based approaches. In this technical review, we identified papers published on applying ML methods focusing on DL models for analyzing both imaging- and sequencing-based SRT data up to September 2023. We present a comprehensive overview of the concepts, tasks, DL models and associated findings in SRT data analysis, with detailed information on current SRT datasets, evaluation metrics and results. Our review aims to be a comprehensive reference for future applications of DL in SRT data analysis and the development of innovative methods. Figure 1 summarizes various SRT methods related to the image-based and sequencing-based approaches.

## OVERVIEW OF COMMON DL MODELS FOR SRT DATA ANALYSIS

**Figure 2:**
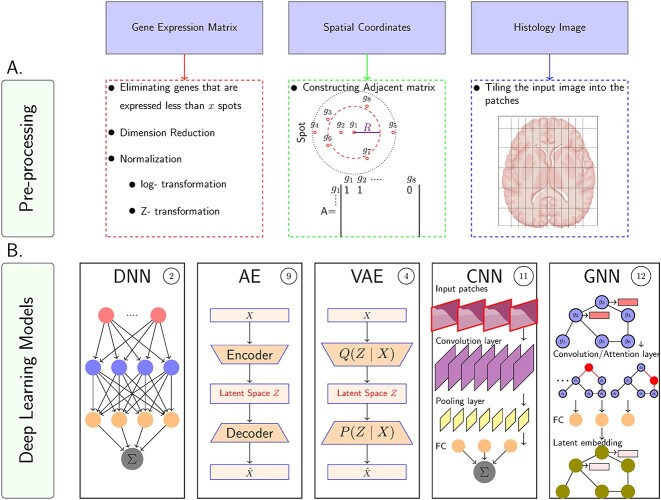
Graphical representations of the **A. Pre-processing step:** For the gene expression matrix, preprocessing includes the elimination of genes with expression below a certain threshold across a specified number of spots, followed by dimension reduction to simplify the data, and normalization, which involves log-transformation and Z-transformation to standardize the expression levels. In parallel, spatial coordinates are utilized to construct an adjacency matrix that captures the spatial relationships between different spots within the tissue—each spot is represented by a node, and the edges reflect the spatial proximity to neighboring spots. For the histology image preprocessing, the original image is partitioned into patches, which allows for a detailed analysis of the tissue’s histological features by breaking down the image into manageable segments for further computational processing. **B. Surveyed DL models in SRT:** These methods include DNN, deep AE, VAE, CNN and GNN. The circled numbers adjacent to each model’s name indicate the total number of papers that have employed the respective model up to September 2023.

In SRT exploration, different supervised and unsupervised learning methods are employed for various tasks. Supervised learning is used for gene prediction and cell segmentation, while unsupervised learning is applied to clustering, gene expression imputation and dimension reduction. Table 1 provides a brief explanation of DL models used for SRT data analysis, including deep neural networks (DNNs), autoencoders (AEs), variational autoencoders (VAEs), convolutional neural networks (CNNs) and graph neural networks (GNNs)(For more detailed information regarding DL models, refer to [30]). Figure 2 represents the surveyed DL models and pre-processing approaches for gene expression matrices, spatial information and histology images. The mentioned models can be categorized as sequential models, generating a sequence of hidden states as a function of the previous hidden state. The problem of sequential mechanism is hindering parallelization within training examples, which becomes critical at longer sequence lengths in larger data sizes, leading to memory constraints [31]. Recently, attention mechanisms (HA) have been developed to reduce the restriction of sequential computation by designing dependencies without considering their distance in the input or output sequences. Additionally, the multi-head attention mechanism (MHA) or transformer [31] is a robust model architecture, allowing more parallelization in DNN-based methods. Since the reviewed papers propose different architectures of DL models and various loss functions, we will explain their topologies in Section 3.

**Table 1:** Summary of DL models and formulas

**Model**	**Explanation**	**Formula**	**Denotation**
DNNs	Feed-forward neural networks with multiple hidden layers and activation functions. Approximate nonlinear transformations for specific goals.	$y_{n} = f(x_{n-1} \cdot w_{i} + b)$	$y_{n}$ : output of neuron $n$, $x_{n-1}$: output of neuron $(n-1)$, $w_{i}$: weight of neuron $i$, $b$: bias,$f$: activation function
AEs	Deep generative models for dimensionality reduction. Encode input data into latent variables and reconstruct input data.	$Z = E_{\theta }(X), \, \hat{X} = D_{\phi }(Z)$	$Z$ : latent variable, $E_{\theta }$: encoder network, $\hat{X}$: reconstructed input, $D_{\phi }$: decoder network
VAEs	Encode inputs as distributions over the latent space. Learn latent features through multi-layer neural networks.	$Z \sim q_{\theta }(Z | X), \, \hat{X} = D_{\phi }(Z)$	$Z$ : latent variable, $q_{\theta }$: conditional distribution, $\hat{X}$: reconstructed input
CNNs	Supervised models for image processing. Extract features from multidimensional input data using convolutional and pooling layers.	$S = X * W$	$S$ : feature map, $X$: input matrix, $W$: kernel
GNNs	Generalized models for graph data processing. Aggregate and transform node information through various network architectures like graph convolution network (GCN) and graph attention network (GAT).	$H^{(l+1)} = f(H^{(l)}, A)$	$H$ : hidden layer, $l$: layer, $A$: adjacency matrix, $f$: function that aggregates and transforms the hidden state of nodes.

## SURVEY OF DL MODELS FOR SRT ANALYSIS

**Figure 3:**
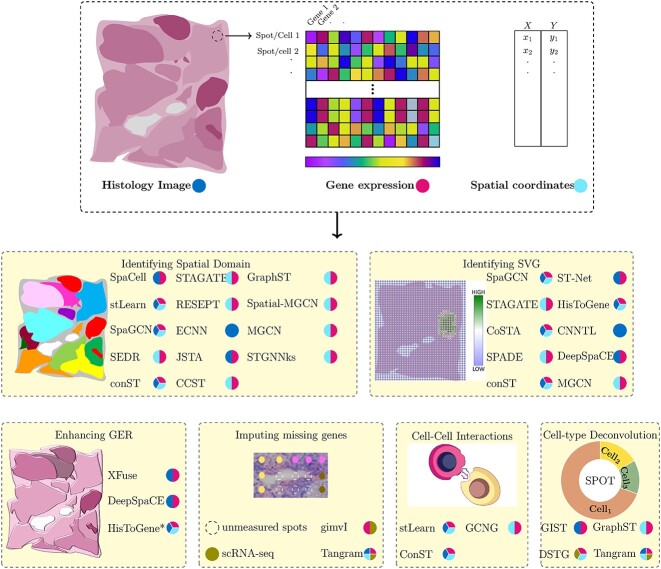
SRT and its six sub-categories with corresponding applications. SRT combines gene expression profiling with spatial information in tissues. Gene expressions are measured in spots, accompanied by high-resolution histology images of the same tissue section. The resolution of spots varies based on the SRT technique, ranging from cellular to sub-cellular levels. DL methods have been employed to analyze SRT data in the following domains: (1) Identifying spatial domains, (2) Identifying SVGs, (3) Imputing missing genes, (4) Enhancement of GER, (5) Cell–cell interactions and (6) Cell-type deconvolution. Each model in the figure represents the SRT data used (blue: histology image, red: gene expression, cyan: spatial information).

We review 26 DL methods used in analyzing SRT data, categorized into six sub-categories: spatial domain identification, spatially variable genes (SVGs), missing gene imputation, enhancement of gene expression resolution (GER), cell–cell interactions and cell-type deconvolution. Figure 3 provides an overview of each sub-category and the corresponding DL methods. Supplementary Tables S1 and S2 present metrics utilized for each category and the datasets, respectively. Figure 3 illustrates the six sub-categories of SRT and their applications.

### Identifying spatial domain

Spatial domain identification is a crucial step in spatial transcriptomics analyses, involving the recognition of spatially coherent areas with consistent gene expression and histology. Various platforms exist for spatial transcriptomics, with some producing both tissue images and gene expression data such as slide-seq [32]. Most approaches rely on clustering methods using gene expression features alone to characterize cell types (i.e. Seurat [33]). Traditional ML techniques like the hidden Markov random field (HMRF) model [34] and Bayesian models like BayesSpace [35] have been employed to incorporate tissue heterogeneity and spatial information. Single-Cell Microscopy Empirical Bayes [36] is also used for spatial domain identification which employs techniques like empirical Bayes and expectation-maximization (EM) to predict labels. Liu *et al*. [37] have identified that the common practice of sequentially performing dimension reduction and spatial clustering is not always accurate. Consequently, they introduced DR-SC, a method that simultaneously addresses dimension reduction and spatial clustering within a single joint model framework. This approach primarily utilizes a probabilistic and hierarchical model, centered around an HMRF model, to efficiently derive low-dimensional embeddings. However, these ML approaches make assumptions about the data-generating process and may not be suitable with limited experimental control.

**Figure 4:**
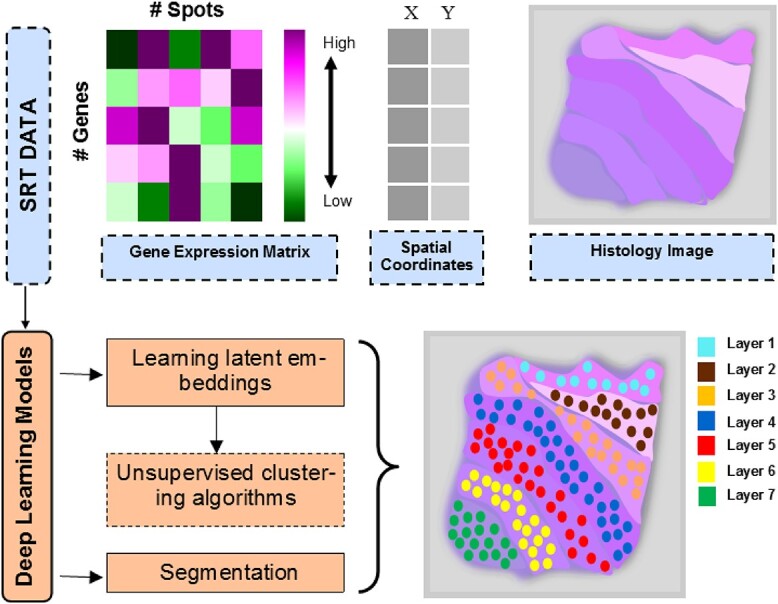
**Identifying spatial domains with DL algorithms on a synthetic tissue.** Mainly, the reviewed papers leveraged deep models to learn latent embedding and then pass them into the unsupervised clustering algorithm; or the papers leveraged DL models to segment the spatial domains.

DL models have gained popularity for analyzing high-dimensional spatial transcriptomics data, especially in sequencing-based methods. DL methods such as SpaCell [38], stLearn [39], SpaGCN [40], SEDR [41], STAGATE [42], RESEPT [43], ECNN [44], JSTA [45], conST [46], CCST [47], GraphST [48], spatial-MGCN [49], MGCN [50] and STGNNKs[51] utilize spatial data and histology images for spatial domain identification. A summary of these DL models is shown in Figure 4, with detailed explanations available in the supplementary material.

Tan *et al*. [38] developed a DL model called SpaCell that combines gene expression data and tissue images for cell-type clustering. The model preprocesses images and count matrices separately, dividing the images into $299\times 299$ pixel tiles and normalizing them. The gene counts are mapped to each spot on the images. The model utilizes ResNet50 [52], a pre-trained CNN, to extract meaningful features from each tile. These features, along with the corresponding gene counts vector, are fed into two AE networks, and the resulting layers are merged to create a latent embedding layer. K-means clustering is then applied to this layer. For disease-stage classification, SpaCell employs the same images and a two-layered DNN model to analyze pixel features and gene count matrices, providing probabilities for four disease stages. However, SpaCell has limitations: it does not utilize spatial information in the embeddings and relies on a pre-training model trained on non-histology images, potentially leading to uninformative outcomes.

Inspired by SpaCell, Pham *et al*. [39] proposed stLearn, which optimizes the integration of gene expression measurements, spatial distance and tissue morphology in spatial transcriptomics. stLearn normalizes gene expression matrices using histology images, called SME normalization (Eq 1), considering neighboring spots with similar gene expression and morphology distance ($MD$). 


(1) \begin{align*}& GE_{i}^{\prime}=GE_{i}+\frac{\sum_{j=1}^{n}GE_{j}.MD_{ij}}{n},\end{align*}

where $GE_{i}^{\prime}$ is the normalized gene expression spot $S_{i}$. $GE_{i}$ and $GE_{j}$ are the raw gene expression for spot $S_{i}$ and its $n$ neighbor spots $S_{j}$. It employs global and local unsupervised clustering, using PCA or UMAP [53] methods, and a k-means clustering applied to a KNN graph constructed based on Euclidean distance. Despite its advances, stLearn uses an unrelated pre-training dataset and a fixed radius for identifying neighboring spots. In contrast, Hu *et al*. [40] developed SpaGCN to link the spatial domain with biological functions, integrating SRT data types using a GCN. SpaGCN constructs an undirected graph ($G(V, E$) where each node’s feature is the gene expression at each spot $\in $  $V$, and the edge’s value ($w$) between two spots $v_{1}$ and $v_{2}$ is determined via spatial coordinates and histological features (Eq 2). 


(2) \begin{align*}& w(v_{1},v_{2})=\exp\left(-\frac{d(v_{1},v_{2})^{2}}{2l^{2}}\right),\end{align*}

where $d(v_{1},v_{2})$ is the Euclidean distance between the two spots and $l$ is a hyper-parameter.

Unlike stLearn, SpaGCN incorporates all spots simultaneously for gene expression aggregation. It employs PCA to reduce the dimensionality of the gene expression matrix and utilizes a GCN for node clustering. However, using RGB channels for dimensionality may yield inaccurate results due to image noise. On the other hand, Fu *et al*. [41] proposed SEDR as an alternative to SpaGCN, highlighting that the integration of histology images and spatial information in SpaGCN is oversimplified. SEDR employs an AE to learn a low-dimensional latent representation of gene expression and incorporates spatial data using the variational graph autoencoder (VGAE) model. The resulting embeddings are concatenated into the final latent representation for spatial clustering. While SEDR excludes histology images, both SpaCell and stLearn have demonstrated the advantages of including them, particularly in addressing tissue heterogeneity.

Dong *et al*. [42] then proposed STAGATE, a graph attention (GAT) AE that corrects predefined similarity measurements in SEDR, integrating spatial data and gene profiles to identify spatial domains in SRT data. In preprocessing, tissue-external areas are removed, and log-transformed gene expressions serve as input. STAGATE’s novelty lies in its adaptive construction of a spatial neighbor network via a standard adjacency matrix with spatial data, radius as a predefined parameter, and through GAT using a pre-clustered gene expression matrix. These modules can be alternately chosen as input for the graph attention layer. STAGATE’s encoder consists of two neural layers, the first adopted to the attention layer. The two layers can be obtained as 


(3) \begin{align*} h_{i}^{1}=\sum_{j\in S_{i}}att_{ij}^{1}\sigma(W_{1}h_{j}^{0}),(layer 1)\end{align*}

(4) \begin{align*} h_{i}^{2}=\sigma(W_{2} h_{i}^{1}),(layer 2),\end{align*}

where $h_{i}^{1}$ is the input gene expression spot $i$, $W$ is the trainable weight matrix, $S_{i}$ is the neighboring set of spot $i$, $\sigma $ is the nonlinear activation function and $att_{ij}$ is the output of the graph attention layer (refer to the supplementary data). It then applies mclust [54] and Louvain clustering on labeled and unlabeled data’s learned features, respectively. Despite STAGATE’s success, its reliance on a predefined radius parameter for identifying neighboring spots is a limitation.

Chang *et al*. [43] criticized SpaGCN [40] and stLearn [39] for lacking spatial information in tissue architecture. They introduced RESEPT, a DL method that reconstructs and segments RGB images from spatial transcriptomics. RESEPT employs a graph AE with a GCN to transform transcriptomics data into a three-dimensional RGB latent space. It integrates a pre-trained ResNet101 backbone network and utilizes a decoder module. However, RESEPT’s fixed neighbor count in the adjacency matrix limits its learning ability and introduces parameter bias.

Chelebian *et al*. [44] proposed ECNN, an adaptation of ensemble CNN [55], to extract holistic features from histology images and transcriptomics signatures. ECNN utilizes $30$ Inception $V3$ [56] models for prostate image classification, generating ensemble latent feature vectors. UMAP downscales the vectors for unsupervised clustering, reducing dimensions to three for visualization. A relative mean intensity matrix confirms genetic relevance. Despite pre-training on related data, ECNN does not consider spatial information in SRT data.

Littman *et al*. [45] responded to the limitations of RNA hybridization-based methods by introducing JSTA, a DL-based EM approach for enhanced RNA hybridization image segmentation. JSTA uses two inputs: the gene expression level of cells and pixels, described by matrices $E_{c}$ and $E_{p}$. The watershed algorithm is initially applied to $E_{p}$ for segmentation. A three-layer DNN is used on $E_{c}$ to link each gene to the cell type with higher likelihood. Another DNN is trained on $E_{p}$ to get each pixel’s cell type probability. These steps form the E-step in the EM algorithm. The M-step involves applying the trained pixel classifier to border pixels for reclassification, followed by updating the image segmentation and cell classifier. The cross-entropy loss function is used in both classification models to minimize error. The process repeats until convergence, improving optimization. However, JSTA lacks generalizability as it is specific to RNA hybridization-based methods. Zong *et al*. [46] presented conST, a user-friendly multi-modal contrastive learning framework (in preprint status as of 10 December) addressing challenges in SRT research. It incorporates gene expression, spatial and morphology information to learn low-dimensional embeddings for clustering. The AE model initializes a general encoder $\varepsilon $ in pre-training, while contrastive learning is used in the main training stage. conST employs a pre-trained masked AE [57] for morphological features, a deep AE for gene expression embedding and VGAE for spatial information. The combined embeddings maximize mutual information. However, conST requires parameter tuning and utilizes non-histology datasets for pre-training. Li *et al*. [47] introduced a method called CCST, utilizing GCNs to analyze spatial gene expression data. CCST combines gene expression profiles with spatial information to enhance cell clustering and subtype discovery. It involves encoding spatial data into matrices, using a hybrid adjacency matrix and a single-cell gene expression profile matrix. These matrices are processed through Deep Graph Infomax [58] networks to calculate cell embedding vectors, integrating both spatial structure and gene expression. The CCST approach’s two main drawbacks are its high computational demands, stemming from the utilization of four graph layers, and its challenges in accurately aligning with pathological annotations. GraphST [48] introduces an approach leveraging self-supervised learning (SSL) with a contrastive loss function. This method aims to generate a corrupted gene expression matrix by shuffling the feature matrix, intending to widen the distance between the original and the corrupted matrices. A graph, constructed with the three nearest neighbors, integrates two GCN layers within the AE framework. The mclust clustering algorithm is subsequently applied to the decoder’s output, utilized as the latent space. However, using the decoder output as the latent space might introduce limitations such as the potential loss of essential data features and sensitivity to noise, with these issues being contingent upon the decoder’s quality. Furthermore, while GraphST proposes a novel loss function to enhance model learning, it does not provide clear insights into the distinctive impacts of employing SSL on the effectiveness of their approach. Recent papers have introduced multi-view graph learning as a novel approach in this field, aiming to obtain latent embeddings by processing different views of input data within their models. For instance, Wang *et al*. [49] generate two graphs from gene expression and spatial information, fusing these representations to achieve a final embedding, which is then used to reconstruct the gene expression matrix through a Zinb decoder. Similarly, Shi *et al*. [50] construct two graphs employing distinct similarity metrics (Euclidean and Cosine similarity) and utilize an attention mechanism to merge the two resulting embeddings. Nevertheless, it is important to note that multi-view learning can be computationally intensive and sensitive to parameter tuning. This approach might also face alignment challenges between different data views, posing risks of poor generalization to new datasets. Building upon the concept of contrastive learning, Peng *et al*. [51] introduced STGNNks, an AE model encompassing four GCN layers. The model employs a hybrid adjacency matrix, a fusion of an identity matrix and an initial adjacency matrix $A_{0}$ as defined by Eq 5. This matrix enhances the depiction of the spatial distribution of gene expression. The researchers also crafted a corrupted adjacency matrix by randomly eliminating edges. The encoder’s embeddings are derived twice: once using the hybrid matrix ($h$) and once with the corrupted version ($\hat{h}$). These embeddings are then fed to the readout function $S$. The primary goal is to maximize the approximated mutual information between $h$ and the output of the readout function, optimizing the learning of spot-specific embedding features. 


(5) \begin{align*}& A=\lambda \times I+(1-\lambda) A_{0}\end{align*}

### Identifying SVGs

**Figure 5:**
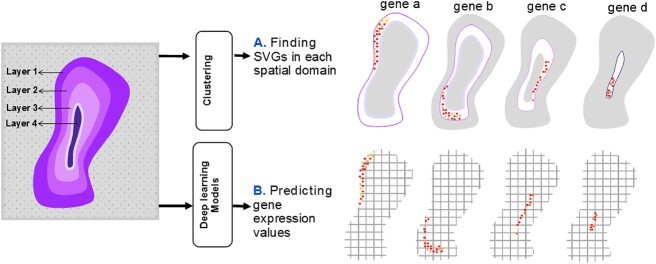
**SVGs detection with DL methods on a synthetic four-layered tissue, including four layers (i.e. spatial domains).** (**A**) Some studies train a deep model to predict marker gene values as a primary task. (**B**) Other studies use ML or statistical methods to detect SVGs in each spatial domain determined by clustering algorithms.

SVG detection in tissue sections is a crucial task that aims to identify spatial expression patterns. While some statistical methods [59–61] have been developed, they overlook tissue taxonomy and miss morphology-related markers. SVG detection approaches can be divided into cluster-based and whole tissue-based methods. Cluster-based methods (e.g. SpaGCN, STAGATE,MGCN and conST) employ statistical tests on spatial domains derived from clustering algorithms, but they fail to detect genes with gradient expression and samples resistant to grouping. Whole tissue approaches are necessary in such cases. ML methods for SVG detection can be spectral or deep-based. Spectral methods, like RayleighSelection [62], calculate the combinatorial Laplacian score (CLS) for each gene, where lower CLS values indicate greater spatial variability. However, spectral methods are computationally intensive and struggle with large datasets. Zhang *et al*. [63] investigated the computational efficiency of recent methods for detecting SVGs. They put forward ScGCO, a novel approach that leverages a probabilistic graph model to encapsulate both statistical and spatial characteristics of the modeled variables in order to accurately identify SV genes. Leveraging H&E-stained histology images, methods combining SVG detection and these images are desirable. DL-based methods, incorporating techniques like *in situ* hybridization and *in situ* sequencing, have demonstrated superiority. DL methods such as CoSTA, ST-NET, SPADE, HisToGene, CNNTL and DeepSpaCE have been proposed for SVG detection in both cluster and deep-based domains. Figure 5 provides an overview of distinguishing SVGs using DL models, as reviewed in this paper.

Hu *et al*. [40] introduced SpaGCN, a methodology devised for identifying SVGs within certain clusters. The approach utilizes the Wilcoxon rank-sum test to pinpoint SVGs, focusing on genes with high expression levels within dispersed domains referred to as ‘metagenes’. By modifying threshold values, selecting foundational genes and managing the addition/subtraction of positive/negative genes, SpaGCN efficiently identifies metagenes specific to target domains. The method also features a sub-cluster option to articulate heterogeneity and demonstrates superior performance compared with SPARK [61] and SpatialDE [60] in SVG detection, as assessed by Moran’s I statistic [64]. Nonetheless, since SpaGCN’s primary function is to train deep models for clustering, the marker genes it identifies might not accurately reflect tissue heterogeneity.

In a similar vein, Zong *et al*. [46] employed conST for the identification of spatial marker genes within clusters, leveraging this as a subsequent task to validate the accuracy of clustering. conST mirrors SpaGCN’s approach, applying it to the latent embeddings derived from its principal algorithm, and exhibits enhanced performance in Moran’s I evaluations conducted on the spatialLIBD dataset [65], especially at the boundary of the white matter layer.

Another model, STAGATE, also aims to identify SVGs within spatial domains, albeit without being specially trained for this purpose. Like SpaGCN, it identifies SVGs that do not necessarily correlate with the tissue morphology. STAGATE implements the Wilcoxon test from SCANPY [66] to spotlight SVGs within each spatial domain. When tested on the Slide-seqV2 dataset derived from mouse olfactory bulb tissue, STAGATE identified a greater number of genes within smaller tissue structures compared with the SPARK-X algorithm.

Finally, MGCN [50] also identifies SVGs following the procedure outlined in SpaGCN and contrasts its performance with both SpaGCN and SpatialDE using Moran’s I statistic test. The results indicate that MGCN can identify a larger number of SVGs than the aforementioned methods. Instead, Xu *et al*. [67] proposed CoSTA, a cluster-based approach which employs an unsupervised CNN to learn spatial relationships between genes using pixel position information from spatial transcriptomic images. Its pre-processing includes pixel binning, gene matrix normalization [60] and scaling. The method consists of two steps: clustering and neural network training. Initially, CoSTA feeds normalized images into a ConvNet, composed of three convolution boxes, each containing convolution, batch normalization and max-pooling layers. Post clustering, labels are generated for ConvNet training. During the second step, a fully connected layer with a softmax activation function is added, providing the probability of $jth$ sample belonging to the $ith$ cluster by an auxiliary target distribution (Eq 6). 


(6) \begin{align*}& q_{ij}=\frac{{p_{ij}^{2}}/f_{i}}{\sum_{i=1}^{N}{p_{ij}^{2}}/f_{i}},\end{align*}

where $N$ is the total number of clusters and $f_{i}=\sum _{j=1}^{M}p_{ij}$, and $p_{ij}$ is obtained through Eq 7. 


(7) \begin{align*}& p(y=i\mid x)=\frac{e^{1/d_{i}}}{\sum_{i=1}^{N}e^{1/d_{i}}},\end{align*}

where $d_{i}$ is Euclidean distances between sample $i$ to cluster centroids $c_{i}$. This layer is only used during training and discarded subsequently. CoSTA outperforms SpatialDE and Spark in identifying gene similarities in the MERFISH dataset. It effectively identifies spatial pattern-dependent genes in Slide-seq data. However, CoSTA requires extensive parameter tuning and is assessed only on high-resolution SRT data. Meanwhile, ST-Net [68] combines spatial transcriptomics with histology images to predict high-resolution gene expression in breast cancer patients. It utilizes a pre-trained DenseNet-121 CNN on ImageNet [69], achieving low mean square error and high Pearson’s correlation on a breast cancer spatial transcriptomics dataset. However, ST-Net underutilizes available spatial data. Refer to the Supplementary material for more details.

Bae *et al*. [70] proposed SPADE, a CNN model that combines gene expression data with image patches to detect SVGs. SPADE utilizes VGG-16 features and PCA for DR. Normalized genes (obtained by Limma [71]) are fitted to PCA-processed image features through linear regression. Genes are ranked based on their correlation with PCA values, and spatial marker genes are selected using a false discovery rate threshold and variance explained by principal components. SPADE successfully identified tissue-specific markers in breast cancer, olfactory bulb and prostate cancer datasets. However, Marker gene identification is influenced by spot density and distance. Pang *et al*. [72] introduced HisToGene (in preprint status as of 10 December) as a solution to the limitations of ST-Net, which does not fully utilize spatial information in its CNN model. HisToGene incorporates an AE with an attention-based mechanism to predict gene expression values, taking into account spatial location and histology images. The model undergoes pre-processing steps such as gene removal, UMI count normalization and transformation to a natural log scale. HisToGene extracts patches from histology images, creating a new matrix that considers variations in spot numbers within tissues, analogous to sentence lengths in natural language processing (NLP). The encoder encodes the new image matrix and spatial coordinate through a single layer, and the sum of these encoding matrices forms the final embedding matrix. Multi-head attention layers, consisting of eight layers and 16 attention heads, are then applied (see [31] for attention model details). HisToGene consistently demonstrated superior correlation compared with ST-Net in its evaluation on high-resolution SRT data.

Abed-Esfahani *et al*. [73] proposed CNNTL, a CNN-based method with contrastive loss, to embed gene expression patterns from human brain images in the ISH method. CNNTL was designed as an alternative to classification-based approaches and employed triplet loss during training. It achieved a rank-1 accuracy of 38.3% on the Cortex dataset, outperforming single ResNet and random models. However, CNNTL’s application is limited to a small subset of genes in brain layers and was pre-trained on an unrelated dataset.

Monjo *et al*. [74] introduced DeepSpaCE, a CNN model specifically tailored for *in situ* capturing technology in Spatial Transcriptomics, with a focus on oncology. DeepSpaCE utilizes a VGG16 network to predict the expression of 24 genes, including breast cancer markers. When evaluated on a human breast cancer dataset, DeepSpaCE showed a correlation coefficient of 0.588 between measured and predicted values. However, DeepSpaCE’s capacity is limited to predicting a specific number of genes.

### Imputing missing genes

**Figure 6:**
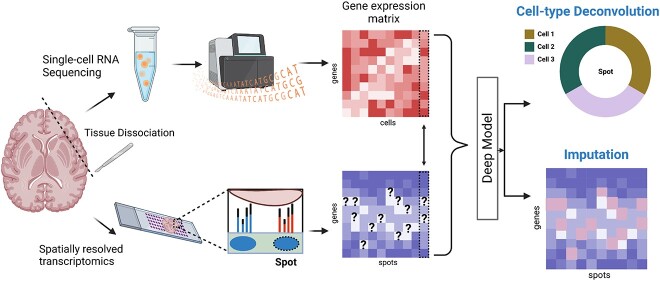
**Imputing missing genes and cell-type deconvolution with DL models on a synthetic tissue.** This figure illustrates the use of DLmodels to impute missing genes and decompose cell types in a synthetic tissue. While sequencing-based techniques only capture spot-specific transcriptomes, single-cell sequencing covers all genes but loses spatial information due to tissue dissociation. By integrating SRT and scRNA-seq data from identical tissues, DL models can detect unmeasured genes and determine cell proportions in each spot. (Created with BioRender.com).

scRNA-seq provides detailed gene expression profiles but lacks spatial context [75, 76]. Alternatively, Spatial Transcriptomics retains spatial context, but its resolution is limited. To address these limitations in each technology, recent studies proposed integrating scRNA-seq and SRT to predict unmeasured genes [77–80]. ML methods, such as LIGER [77] and SpaGE [78], use joint dimension reduction techniques, like NMF and PCA, respectively, followed by linear models for joint embedding. GimVI, a joint non-linear model, uses deep generative models for domain adaptation [79]. However, as Shengquan *et al*. [80] noted, these methods often rely on shared genes between the datasets and potentially misleading metrics, like the Spearman correlation coefficient. Therefore, they proposed an AE model, stPlus, which uses the k-NN algorithm for gene prediction. stPlus outperformed SpaGE, Seurat, Liger and gimVI across four clustering metrics: AMI, ARI, Homo and NMI (Supplementary Table S1). In the following text, we focus on the DL models and investigate the three DL models in detail. Figure 6 represents the process of gene imputation along with cell type deconvolution (refer to the next section) in SRT data.

Lopez *et al*. [79] proposed gimVI (in preprint status as of 10 December), which employs a VAE model to analyze gene expression matrices from scRNA-seq and SRT experiments. It distinguishes between the two by utilizing a binary variable and generates a latent vector to represent cell types. gimVI incorporates a K-NN algorithm in the latent space to impute missing genes. It demonstrated superior imputation performance compared with Liger and Seurat, although the results can be influenced by the choice of K and evaluation limited to a fraction of genes in the SRT dataset.

Biancalani *et al*. [81] developed Tangram, which uses DL for mapping spatial information into scRNA-seq data and aligning histological data to anatomical positions. It employs nonconvex optimization to update the alignment based on an objective function that compares cell-density distributions and evaluates gene expression. While primarily designed for spatial map reconstruction, Tangram also performs gene imputation effectively. No quantitative comparison with other imputation methods was provided in the original paper. A recent study [82] ranked Tangram as the third-best imputation method, after stPlus and gimVI, noting its longer running time.

### Cell-type deconvolution

In spatial transcriptomics, transcripts are captured at spatial locations or ‘spots’ [83] comprising mixed, low-resolution cells. Spot-level cell composition identification is crucial due to varying cell numbers from tissue heterogeneity or Spatial Transcriptomics technology [84]. Computational methods for this task fall into three categories: inference-based, multivariate analysis/linear algebra-based and DL-based methods. Inference-based methods like Stereoscope [85], RCTD [86], cell2location [87], DestVI [88] and STdeconvolve [89] employ likelihood-based approaches, assuming input data distribution. Multivariate analysis and linear algebra-based methods, such as SPOTlight [90] and SpatialDWLS [91], incorporate both ML and statistical elements, yet have limitations as outlined in Section 1). Meanwhile DL-based methods like GIST, Tangram and GraphST estimate cell-type proportions with DL models. Even though some methods such as VAE are probability-based, they are still categorized as DL methods in this paper. However, these methods also have real-world application limitations.

Song *et al*. [92] proposed DSTG, a semi-supervised graph convolution network method for decomposing cell mixtures in SRT data. It creates pseudo-ST data from scRNA-seq data, constructs a linked graph and utilizes a GCN network with three convolution layers to predict cell type proportions. DSTG outperforms the SPOTlight [90] method on synthetic and real SRT datasets. However, comparing pseudo-ST and real-ST using Euclidean distance may not provide a fair comparison.

Biancalani *et al*. [81] addressed the limitations of inference-based deconvolution methods that overlook spatial information, leading to flawed cell-type detection. They developed Tangram, which performs deconvolution on ST/Visium technology, specifically low-resolution SRT data. Tangram calculates cell counts through initial segmentation and maps cell-type ratios consistently in multiple datasets. However, the reliance on prior knowledge about cell numbers for segmentation may be a drawback, especially in high-density tissues like tumors [81].

Zubair *et al*. [93] proposed GIST, a joint model integrating SRT and image-derived data to enhance cell-type deconvolution. GIST utilizes DL on images to provide preliminary information for cell type identification within a Bayesian framework. A CNN model estimates cell-type abundance, particularly immune cells, by processing JPEG images into encoded TIFF format and generating probability maps. Spot-level probabilities are calculated by weighted summation of overlapping patches. GIST outperformed the base model using only expression data in identifying immune cells in breast cancer pathology. However, comparative evaluations with Tangram and DSTG are needed to determine its relative performance.

Long *et al*. [48] aim to derive a mapping matrix $M$ that indicates the cell percentage in each spot. Initially, they utilize an AE model to obtain the reconstructed cell gene expression matrix $H_{C}$ from sCRNA-seq data and the reconstructed gene expression $H_{S}$ from ST data. Subsequently, they predict the spatial gene expression matrix $H_{S}$ by integrating it with the mapping matrix, expressed as $H_{S}^{^{\prime}}=M^{T} \times H_{C}$. The authors endeavor to learn the mapping function $M$ through a mechanism of augmentation-free contrastive learning.

### Enhancement of GER

**Figure 7:**
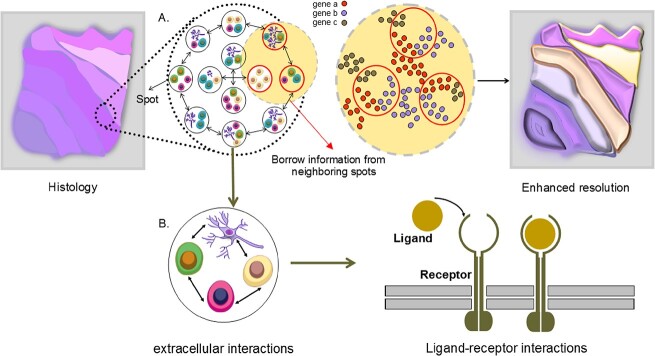
**Enhancing GER and cell–cell interactions in SRT data.** (**A**) Since the distances between spots are different based on the utilized sequencing-based approaches, borrowing information from neighboring spots makes it possible to enhance the GER in empty areas between spots. (**B**) The spatial location of each spot facilitates the understanding of finding ligand–receptor interactions of each cell in SRT data.

Improving GER in SRT data, often limited at the single-cell level, has led to the proposal of various DL methods. These aim to enhance GER in SRT data by borrowing information from neighboring areas to fill gaps between spots (Figure 7). While SRT technologies like Visium and SLIDE-seq [32] provide high-resolution cell morphology information, statistical methods like RCTD, which estimate cell-type-specific gene expression per spot based on deconvolution probability, can be unreliable as their accuracy depends on the deconvolution step. BayesSpace addresses this by dividing each spot into equal-size sub-spots and inferring gene expression, keeping total expression constant. However, the variability in splitting methods can lead to different outcomes, complicating the determination of an optimal solution. Given their ability to integrate multiple data, DL methods utilizing histology images have been proposed to enhance GER, a tactic not utilized by the aforementioned methods.

Bergenstrahle *et al*. [94] developed XFuse, a tool that integrates low-resolution *in situ* sequencing gene expression data with high-resolution histology images to infer high-resolution spatial gene expression. XFuse assumes negative binomial and Gaussian distributions for gene expression data and histology images, respectively. It utilizes a convolutional generator network to map parameters from the latent tissue state. Through variational inference, XFuse estimates the posterior of the latent variable by minimizing the Kullback–Leibler divergence. Histology images are mapped to the latent tissue state using a convolutional recognition network. XFuse outperformed a method using non-missing neighbors’ information and successfully revealed distinct patterns in mouse olfactory bulb and human breast cancer datasets. It exhibited lower median RMSE, accurately predicted unseen samples, and showed better prediction of gene expression patterns compared with *in situ* hybridization data. However, XFuse is limited to detecting genes with spatial patterns resembling the histology images.

Pang *et al*. [72] extended their previous work on HisToGene to develop HisToGene*, a super-resolution gene expression prediction method using dense histology image patches. HisToGene* applied the trained model to estimate spot-level resolution gene expression by treating spots as sentences in NLP. Sub-patches covering four patches each were created to predict higher resolution gene expression compared with the original spot. The results showed that HisToGene* predictions had higher correlations with observed spot-level gene expression in 19 sections, while HisToGene showed superior correlations in six sections. HisToGene* predicted gene sets demonstrated a direct link between thyroid hormones and breast cancer risk [95], indicating the presence of more biologically significant information.

Utilizing super-resolution techniques for spatial gene expression and tissue section imputation, Monjo et al. [74], employed semi-supervised learning (SSL) to enhance prediction performance. DeepSpaCE uses a trained model to estimate unmeasured genes in images with inadequate gene expression. The method was tested on a human breast cancer dataset with various tissue sections, using certain sections for training and others for testing. The model, acting as a ‘teacher’ in SSL, improved Pearson’s correlation coefficients (PCC) between actual and predicted expression when using unlabeled data from other sections. Applying the SSL approach to ImageNet’s cat and dog images and the Cancer Genome Atlas (TCGA) dataset did not yield any improvement.

### Cell–cell interactions

Cell–cell interactions, a crucial extracellular communication process involving ligand–receptor interactions (LRI), is a primary focus in understanding intercellular communication [96]. Existing computational methods often concentrate on intracellular interactions or are limited to small-scale experiments. Spatial transcriptomics, offering gene expression profiles in spatial coordinates within cells, shows promise in predicting LRIs (Figure 7). Giotto [97] is a comprehensive framework for analyzing such data, including a cell–cell interaction module. Statistical methods like Giotto and ML frameworks like MISTy [98] identify cellular niche interactions by modeling expression of markers and generating pairwise distances, respectively. MISTy uniquely identifies interactions within specific regions, aiding marker interaction understanding, but it is computationally intensive. While non-DL models’ performance is impacted by growing SRT data diversity, DL methods can better identify cell interactions in large SRT datasets. Pham *et al*. [39] developed stLearn, a method for analyzing cell type diversity and identifying receptor–ligand interactions (RLIs) in tissue sections. It involves quantifying cell-type diversity and calculating co-expression of ligand–receptor pairs using CellPhoneDB [99]. A significant ligand–receptor pair matrix (CCI matrix) is created, and tissue regions with similar co-expression are clustered. stLearn combines cell density and CCI measures to identify areas with high cell–cell interaction probability. Validated on a breast cancer dataset, stLearn revealed significant interactions between tumor and immune cells.

Yuan and Bar-Joseph [100] proposed GCNG, a graph convolutional network for gene expression, to overcome limitations in extracellular interaction detection from Spatial Transcriptomics data (like Giotto). GCNG takes spatial cell locations and gene expression pairs as inputs, constructing an adjacency matrix based on Euclidean distance. The model maps the matrices’ product to an embedding vector, enabling investigation of interactions between indirectly linked cells. GCNG outperformed other models, achieving high AUROC/AUPRC values. However, its predefined distance criteria for neighbor cell selection may introduce biases. Lastly, conST leveraged the advantages of clustering, SVG detection, and trajectory inference. This method identified target receptors on breast cancer cells and analyze their microenvironment in IDC regions. Initially, it derives latent features from the dataset and segregates them into 20 clusters. It then identifies three clusters containing significant lesion areas and applies trajectory inference for pseudotime ordering. The SVG detection algorithm is used to identify marker genes responsible for the tumor microenvironment. Finally, cross-cluster CCI analysis is performed using TraSig [101], and within-cluster analysis is carried out by label transfer from Seurat to identify active ligand–receptor pairs. The results showed conST’s capability to detect IDC, DCIS and edge tumor cell regions and active L-R pairs within IDC regions.

## DISCUSSION AND FUTURE LOOK

In this study, we delve into the strengths and limitations of DL methods for analyzing spatial transcriptomics data. Our in-depth technical overview provides insights into the performance of each method. The analysis of SRT data is a swiftly progressing area featuring a wide array of methods and applications across various categories. To ensure an unbiased comparison of these methods regarding their applicability, it is crucial to explore independent benchmarking techniques. Such an approach allows for an equitable assessment of these methods, especially considering how the benchmarks themselves are executed. Consequently, we have compiled a list of the latest benchmarking methods, categorized accordingly, in Table 2.

**Table 2:** Independent benchmarking links for each category

**Category**	**Link**
Identifying spatial domain	Teng Liu *et al*. [102]
	AndrewCheng [103]
Identifying SVG	Charitakis *et al*. [104]
Cell-type deconvolution	Li *et al*. [105]
	Yan and Sun [106]
Gene expression imputation	Avsar and Pir [82]
Cell–cell interaction	Liu *et al*. [107]

We also comprehensively summarized the reviewed DL algorithms proposed for SRT data analysis in Table 3, providing an opportunity to readers to quickly overview each method.

**Table 3:** Deep learning algorithms

**Algorithm**	**Models/Method**	**Advantages**	**Limitations**	**Data Type** ^*^	**Codes**
**Identifying spatial domain**					
SpaCell [38]	CNNAEDNN	Can incorporate the three types of spatial transcriptomics data:histology,imaging, and gene expression	Pre-trained on the ImageNet which is unrelated to SRT	Slide-seq	https://github.com/BiomedicalMachineLearning/SpaCell
SpaGCN [40]	GCN	Can incorporate the three types of the spatial transcriptomics data:histology,imaging, and gene expression. can detect SVGs.	Using the RGB channel to analyze the histology images may not be appropriate in noisy images. Inadequate reasons to show the influence of the histology images in ST methods.	smFISHSlide-Seq	https://github.com/jianhuupenn/SpaGCN
stlearn [39]	CNN	Combine three various type of data	Lack of quantitative comparison	10x Genomics	https://github.com/petersaj/histology
SEDR [41]	GCNAEDNNVGAE	Can learn the low-dimension representation of gene expression jointly with embedding spatial information	Defining the similarity between spots before training and did not consider the learning strategy	10x GenomicsStereo-seq	https://github.com/HzFu/SEDR
STAGATE [42]	GAT	Can adaptively learn the similarity between spots and construct SNN based on the adjacency matrix and cell-type aware module. First method for constructing a 3D pattern with spatial information.	Rely on a radius parameter to determine the adjacency matrix	10x VisiumSlide-seq Slide-seqV2Stereo-seq	http://spatial.libd.org/spatialLIBD
RESEPT [43]	GCNCNNAE	Can reconstruct RGB image from gene expression or RNA velocity from SRT data.	Use the fixed number of neighbors to build the input graph.	10x Visium	https://github.com/OSU-BMBL/RESEPT
ECNN [44]	CNN	Can pre-train CNN on the relative dataset instead of using ImageNet.	The proposed model is blind to SRT data	10x Visium	-
JSTA [45]	DNN	Can enhance the cell segmentation at the cell borders by jointly using cell-type expression patterns and RNA hybridization-based spatial transcriptomics.	The method was not compared with the other ML methods. It uses parameter tuning empirically to identify cell borders.	MERFISHosmFISH	https://github.com/wollmanlab/JSTA
conST [46]	GCNVGAEAE	It uses multi-modal contrastive learning that can be utilized in both SRT categories.	High parameters tuning.	seqFISHMERFISH10x VisiumStereo-seq	https://github.com/ys-zong/conST
CCST [47]	GCNAE	Introduces a hybrid adjacency matrix and provide biological interpretation of clustering outcomes	High computational cost	SeqFISHMERFISH10x Visium	https://github.com/xiaoyeye/CCST. https://doi.org/10.5281/zenodo.6560643
GraphST [48]	GCNAE	Introduces a self-supervised learning approach with a contrastive loss function	lack of clarity on the effectiveness distinctions when employing SSL and sensitivity to noise dependent on decoder quality.	Slide-seqV2MERFISH10x VisiumStereo-seq	https://github.com/JinmiaoChenLab/GraphST
Spatial-MGCN [49]	GCNAE	Use Multi-view Graph Convolutional Network	computationally intensive and sensitive to parameters	Stereo-seq10x Visium	https://github.com/cs-wangbo/Spatial-MGCN.
MGCN [50]	GCNAE	Used Multi-view Graph Convolutional Network	computationally intensive and sensitive to parameters	Stereo-seq10x VisiumosmFISHseqFISH	https://github.com/sxj204/stmgcn.git.
STGNNks [51]	GCNAE	Enhanced spatial gene depiction using a hybrid adjacency matrix.	Relies on accurate hybrid adjacency matrix construction	10x Visium	https://github.com/plhhnu/STGNNks
**Imputing Missing Genes**					
gimVI [79]	VAE	Can jointly use scRNA-seq and spatial transcriptomics to impute missing genes in SRT data.	Evaluate the model on the small part of genes, which obtain relatively low Spearman correlation.	osmFISHstarMAP	https://github.com/scverse/scvi-tools
Tangram [81]	CNN	Can provide an automatic pipeline for locating histology data on an anatomically annotated CCF.	It requires a CCF, which is available for a few organs related to the mouse brain.	In situ hybridization	https://github.com/broadinstitute/Tangram
**Identifying SVG**					
CoSTA [67]	CNN	Learn broader spatial patterns	High parameters tuning	In Situ Hybridization	https://github.com/rpmccordlab/CoSTA
SpaGCN [40]	Wilcoxon rank-sum test	using strict criteria to ensure only genes with significant spatial expression variations are selected.	The outcomes can be significantly influenced by the output of the clustering process.	smFISHSlide-Seq	https://github.com/jianhuupenn/SpaGCN
STAGATE [42]	Wilcoxon rank-sum test	Can dynamically discerns the similarities among spatial spots, enabling a refined and precise identification of genes with spatially influenced expression profiles.	The outcomes can be significantly influenced by the output of the clustering process.	10x VisiumSlide-seq Slide-seqV2Stereo-seq	http://spatial.libd.org/spatialLIBD
ST-Net [68]	CNN	Can link gene expression with visual features. Can generalize in the other breast cancer spatial transcriptomics data due to the promising results in external validation.	Spatial information is not utilized in the model.	In situ sequencing	https://github.com/bryanhe/ST-Net
SPADE [70]	CNN	Can identify maker genes associated with morphology. Can use gene ontology to find the relationship between obtained principal components from image features and biological processes.	The obtained marker genes can be significantly affected by the spot’s density and the distance between spots.	10x Visium	https://github.com/mexchy1000/spade
HisToGene [72]	MHA	Can join histology images with spatial information to predict gene expression;first paper for high-resolution gene expression prediction.	May struggle with noisy or low-quality histology images, impacting the accuracy of spatial variable gene identification.	10x Visium	https://github.com/maxpmx/HisToGene
CNNTL [73]	CNN	Can evaluate the CNN model on the ISH images to predict gene expression and utilize triplet loss to overcome the lack of enough labels for each gene.	The method is limited to a small fraction of genes in brain layers. The model pre-trained on the datasets unrelated to SRT data.	In situ hybridization	https://github.com/PegahA/Human_Brain_ISH_ ML
DeepSpaCE [74]	CNN	Use SSL to enhance the CNN model. Perform super-resolution and section imputation methods, which reduce the experimental costs.	It predicts only limited genes. The model only can be applied to the images.	In situ capturing	https://github.com/tmonjo/DeepSpaCE
conST [46]	GCNVGAE	leverages GCN and VGAE to effectively identify genes with spatially dependent expression, capturing complex spatial patterns.	Require fine-tuning of parameters for optimal performance	seqFISHMERFISH10x VisiumStereo-seq	https://github.com/ys-zong/conST
MGCN [50]	Wilcoxon rank-sum test	Capturing complex spatial relationships in tissue, which is crucial for accurate SVG detection	May be sensitive to parameter settings, necessitating careful tuning to achieve optimal results	Stereo-seq10x VisiumosmFISHseqFISH	https://github.com/sxj204/stmgcn.git.
STGNNks [51]	SpatialDE [60]	Potentially uncovers more detailed spatial gene expression patterns that broader analyses might overlook.	Heavily relies on the quality of the initial clustering	10x Visium	https://github.com/plhhnu/STGNNks
**Enhancement of GER**					
HisToGene [72]	MHA	Improves the spatial resolution of gene expression predictions by combining detailed histological information with transcriptomics data.	Potentially less effective with low-resolution spatial transcriptomics data, limiting its applicability in some datasets.	10x Visium	https://github.com/maxpmx/HisToGene
XFuse [94]	VAE	Can find a clear pattern of low-resolution SRT data and impute missing genes at high-resolution.	Only detect genes whose spatial patterns are similar to the histology images.	In situ RNA capturing	https://github.com/ludvb/xfuse
DeepSpaCE [74]	CNN	Use SSL to enhance the CNN model. Perform super-resolution and section imputation methods, which reduce the experimental costs.	It predicts only limited genes. The model only can be applied to the images.	In situ capturing	https://github.com/tmonjo/DeepSpaCE
**Cell-type deconvolution**					
GIST [93]	CNN	Can use the image as informative prior information to improve cell-type deconvolution	Did not consider the spatial data. It Needs to tune the hyper-parameter $\lambda $ empirically.	In Situ Sequencing	https://github.com/asifzubair/GIST
DSTG [92]	GCN	Apply semi-supervised graph convolution network to detect cell type deconvolution in ST data. It generates pseudo-ST data by scRNAseq data and provides ground truth for the learning process.	Considering the Euclidean distance between the pseudo-ST and real-ST data to show the similarity between two data is not a fair comparison.	10X Genomics VisiumSlide-seq v2	https://github.com/Su-informatics-lab/DSTG
GraphST [48]	GCNAE	Utilizing a mapping matrix to project scRNA-seq data into spatial transcriptomics space, rather than depending solely on a cell-type composition matrix.	This method might not fully account for the intricate and dynamic nature of cell-type compositions within different spatial contexts.	Slide-seqV210x VisiumStereo-seq	https://github.com/JinmiaoChenLab/GraphST
Tangram [81]	Probabilistic Mapping	flexible integration with various spatial and single-cell datasets	Requires comprehensive single-cell reference data; computational intensity	10x Visium	https://github.com/broadinstitute/Tangram
**cell-cell interaction**					
stlearn [39]	Permutation test	Using spatially-Constrained and Two-level Permutation (SCTP) method to effectively reduces false discoveries in cell-cell interaction analysis by robustly identifying significant ligand-receptor pairs based on spatial context.	Complexity of the method, especially due to the implementation of the two-level permutation test.	10x Genomics	https://github.com/petersaj/AP-histology
GCNG [100]	GCN	Can identify extracellular interaction in SRT data, even in cells without direct relationship.	The model relies on the pre-defined distance criteria for selecting the neighbor cells.	seqFISH+MERFISH	https://github.com/xiaoyeye/GCNG
conST [46]	TraSig [101] and Seurat	Enabling detailed assessment of tumor progression and the risk of spread to neighboring areas.	The CCI method has been exclusively evaluated using breast cancer data from the 10x Visium platform	10x Visium	https://github.com/ys-zong/conST

The Data type column specifies the types of datasets on which the methods listed have been applied, as reported in their respective studies.

To underscore the efficacy of DL methods in processing SRT data, we contrast them with non-DL techniques. It is important to note that the success of many downstream tasks is contingent on the effective functioning of individual components within the overall workflow. For instance, the identification of SVGs hinges on the clustering algorithm, meaning any overlooked biologically relevant feature could hinder downstream analysis. Consequently, we propose incorporating ‘pathway information’ into SRT data via phylogeny-aware clustering techniques [108], which are becoming increasingly prevalent in the analysis of biological datasets. This could involve integrating KEGG-level pathways or other reference assignments into the effect size of a gene. Such an approach helps recognize that the impact of changes in two genes functioning within the same pathway is less significant overall compared with alterations in genes from entirely different pathways.

Despite the abundant information in SRT data, we found that the current techniques do not fully exploit the rich information in SRT data. There is a clear need for robust DL methods that utilize spatial data, scRNA-seq and high-resolution histology image data together. While CNNs have shown promise in analyzing SRT data, challenges arise due to the unique, complex nature of histology images, especially when combined with spatial data. We advocate for proof that features extracted by deep models hold biological significance. For instance, ECNN [44] and ST-NET [68] have visualized features from intermediate or latent vectors. Additionally, CCST [47] has incorporated cell cycle phase identification as a means to verify the biological relevance of clustered cell groups, using differential expression analysis and Gene Ontology (GO) term enrichment. The clusters were mapped to different cell cycle phases, providing a biologically meaningful interpretation of the clustering results. Dealing with large histology images also requires novel methods to unify small patches, akin to words in NLP techniques [72]. Although HisToGene [72] has linked patches through an attention-based model, most techniques do not account for the relationship between patches, leading to batch effect sensitivity in CNN-based models. Future approaches could consider patches as time-series problems, leveraging DL methods on sequential data, like RNN, LSTM [109] and transformers.

Another essential issue in SRT data processing is the batch effects, which amplified by the volume of spatial transcriptomic datasets, remain a significant challenge. While DL methods have been developed to address this in scRNA-seq [110, 111], the problem is more complex in SRT due to spatial dependency and the association with histology images. SEDR [41] and STAGATE [42] represent initial efforts to mitigate batch effects, but neither account for histology images, necessitating methods that evaluate gene expression and histology images together. SRT, allowing analysis of imaging and molecular features, could significantly advance disease diagnosis. Both SpaCell and CNNTL [73] exemplify the use of image and gene expression data in disease classification. As SRT technology evolves and data generation costs drop, its use in routine disease diagnosis could be transformative, particularly when capturing parallel biological variables like sex, race and age. Such inclusion in SRT data could revolutionize disease identification. Pre-processing is another critical step in SRT data analysis, impacting the results significantly. The gene expression matrix generated from sequencing machines constitutes compositional data, describing gene abundance as proportions to other genes within a sample [112]. Instead of residing in Euclidean space, these compositional data lie within a subspace called the simplex [113]. While Aitchison distance is proposed within the simplex, methods like log-ratio transformation map data to real space, making Euclidean distance relevant and preventing data misinterpretation [113]. Although the majority of reviewed techniques use Euclidean distance for spatial coordinates appropriately, applying PCA or clustering algorithms on untransformed data contradicts the compositional data hypothesis. Only 13 methods of those reviewed considered log-transformation on the gene expression matrix. For example, stLearn employs SMEClust normalization, executing PCA and UMAP on normalized genes sans transformation. It is recommended that future work considers gene expression matrix compositionality and explores other transformation and normalization approaches.

SRT data’s gene expression matrix is sparse, presenting significant overdispersion and zero values, which poses challenges for count data modeling. Given that many statistical and DL models, like VAE models, directly engage with count data, understanding gene expression’s overdispersion and zero inflation patterns is crucial. It helps determine whether sparsity arises from platform-based issues necessitating imputation or from tissue location-based gene expression heterogeneity requiring overdispersion and zero inflation management. Poisson or negative binomial models are preferable for most SRT technologies [114], handling overdispersion without additional zero inflation term. Excessive zero count potentially signifies biological variation, where imputation might introduce noise through non-zero values, negatively affecting analysis. We highly recommend reassessing existing imputation methods like gimVI and Tangram, considering these limitations.

All the methods we have mentioned are reference-based, requiring a matching scRNA-seq dataset from the same tissue to estimate cell proportions. Chen *et al*. [115] evaluated the impact of gene-subset selection and the effectiveness of deconvolution methods using both internal and external inference. They found Tangram and DSTG performed best with perfectly matched internal references and that gene selection can impact deconvolution performance. In particular, top cell-type marker genes outperformed highly variable gene subsets for external reference use. Many studies treated dimension reduction methods as error-free techniques to obtain low-dimensional features. However, we suggest future studies to develop a unified loss function for dimension reduction and clustering and evaluate the performance of dimension reduction approaches [37].

In Liu *et al*.’s [107] recent research, the focus was on exploring the impact of spatial distances on CCI analysis. The findings underscored the value of combining various data types, such as SRT and scRNA-seq, to improve the accuracy of CCI predictions. The study highlighted a significant limitation in current ST approaches, particularly the spatial resolution constraints. These limitations tend to cause a mix of gene expression patterns from different cell types within the same spatial location, thereby affecting the precision of CCI predictions. Consequently, there is a necessity to use scRNA-seq data as a reference for discerning cell types in ST-guided CCI studies. The introduction of some SRT technologies, like Stereo-seq [116], which provide single-cell resolution, marks a significant advancement. However, the development of effective downstream analytical methods for technologies like Stereo-seq remains a work in progress. With the ongoing advancements in high-resolution SRT data, there is an expectation for the emergence of more sophisticated CCI tools. These tools would be capable of analyzing single-cell SRT data without relying on scRNA-seq-based approaches, paving the way for more accurate and detailed CCI studies [107].

Assay for Transposase Accessible Chromatin with high-throughput sequencing [117] provides genome-wide chromatin accessibility profiling, and its single-cell variant, scATAC-seq, offers single-cell resolution. Despite several spatial chromatin accessibility profiling advancements [118, 119], existing epigenomic methods lack spatial resolution. VAEs have recently been employed for tasks like gene imputation, such as in gimVI, and understanding the interplay between gene expression and TCR sequence [120]. There is growing interest in integrating multi-omics data, including transcriptional and chromatin landscapes of single cells [121–123]. Therefore, it is suggested that developing DL models for integrating scATAC-seq and spatial ATAC-seq to jointly learn the latent embedding could be innovative [124]. Lastly, the computational demands of DL methods in spatial transcriptomics are significant, as shown by Liu *et al*.’s [102] comprehensive evaluation. This study analyzed the efficiency of various GNN-based approaches in spatial domain identification across multiple datasets. It highlighted the high resource requirements of methods like CCST, which, even with a high-end GPU, faced memory issues with complex datasets like Slide-seqV2 and seqFISH. Similarly, GraphST and conST, integrating multiple neural networks, needed more computational resources, resulting in increased runtimes and memory usage. Also, in another benchmarking study by Cheng *et al*. [103] various ML and DL methods used in spatial transcriptomics were compared for computational efficiency across seven different datasets. SpaCell showed the lowest memory usage, followed by SpaGCN and Seurat. BayesSpace and stLearn, which account for spatial locations, were more memory-intensive. Regarding runtime, Seurat, SpaGCN and Giotto had similar efficiencies, with most methods showing a linear increase in runtime as dataset size grew. However, Giotto runtime significantly increased with larger datasets. These findings underscore the substantial impact that both dataset characteristics and the architectural complexity of GNNs exert on the computational demands of spatial transcriptomics analyses.

Building on the previously discussed challenges and advancements in spatial transcriptomics, particularly those related to computational demands and accuracy, it becomes essential to delve deeper into the specifics of cell segmentation. This aspect is especially critical in image-based techniques within the field. Recent attempts to refine cell segmentation can be broadly divided into two main categories. The first category includes methods that solely rely on imaging, like Watershed algorithms [125] and CellPose [126]. These approaches, while useful, often face limitations due to the inherent noise in images and labels. Furthermore, as they typically focus on nuclei staining, they tend to capture nuclear boundaries more accurately than the actual cell boundaries [127], making them less suitable when transcriptomic data are not incorporated. The second category encompasses methods that integrate spatial positioning of RNA sequences to infer cell boundaries. This group includes innovative techniques such as JSTA, Baysor [128] and GeneSegNet [127]. As the volume of genes captured increases and computational methods continue to evolve rapidly, there is a growing need for efficient, capable methods. These methods must not only handle the large sizes of image and spatial data but also adapt to the intricacies of spatial transcriptomic analysis.

## CONCLUSION

In conclusion, this paper exhaustively reviewed the DL methods for addressing the analysis challenges in SRT data. DL algorithms excel at recognizing complex patterns and processing large, multi-modal data, making them ideal for the increasingly diversified SRT data. Methods were categorized into six tasks: identifying spatial domains, identifying SVGs, imputing missing genes, enhancing GER, analyzing cell–cell interactions and performing cell-type deconvolution. We aim for this review to guide the use of DL in SRT analysis and inspire collaborations to create innovative methods integrating gene expression, spatial information, single-cell data and digital pathology.

**Key Points**

- Spatially resolved transcriptomics is a new technology providing the position of captured expression across the tissue at single-cell level resolution.
- A total of 26 deep learning-based methods are systemically reviewed in this paper and categorized into six main groups based on the tasks and downstream analyses.
- A brief discussion of the current machine learning approaches is presented for each category to assess the advantages of deep learning models proposed for that category in comparison with the traditional machine learning models.
- A unified description of the model and result corresponding to each deep learning model is presented, and the mathematical model is also discussed in the supplementary section.
- Lastly, a comprehensive summary of the deep learning algorithm, evaluation metrics and datasets by each approach is tabulated.

## Supplementary Material

supplementary_information_2-1_bbae082

## Author Biographies

**Roxana Zahedi** is a PhD student in biomedical engineering at the UNSW BioMedical Machine Learning Laboratory. Her research focuses on medical data analysis, machine learning, and deep learning.

**Reza Ghamsari** is also a PhD student at the same laboratory, specializing in single-cell data analysis.

**Ahamdreza Argha**, a member of UNSW Sydney's School of Biomedical Engineering, conducts research in deep learning and health data analysis.

**Callum Macphillamy**, based at the University of Adelaide, specializes in applying machine learning to animal genetics.

**Amin Beheshti** serves as the director of the Macquarie Centre for Applied AI.

**Roohallah Alizadehsani**, affiliated with Deakin University, focuses on health data analytics.

**Nigel Lovell** leads the School of Biomedical Engineering at UNSW Sydney and directs the Tyree Institute of Health Engineering (IHealthE).

**Mohammad Lotfollahi** is a faculty member at the Wellcome Sanger Institute and the CCAIM at the University of Cambridge.

**Hamid Alinejad Rokny**, also from UNSW Sydney's School of Biomedical Engineering, is the director of the UNSW BioMedical Machine Learning Laboratory, specializing in medical AI and digital medicine.
