# Supplementary material for: Deep learning in spatially resolved transcriptomics: a comprehensive technical view
Source: Brief Bioinform. 2024 Mar 13;25(2):bbae082. doi: 10.1093/bib/bbae082 (PMC10939360; doi:10.1093/bib/bbae082)
Supplement: supplementary_information_2-1_bbae082 [file supplementary_information_2-1_bbae082.pdf]

# Supplementary Information: Deep Learning in Spatially Resolved Transcriptomics: A Comprehensive Technical View

Roxana Zahedi, Reza Ghamsari, Ahmadreza Argha
, Callum Macphillamy, Amin Beheshti, Roohallah Alizadehsani, Nigel H. Lovell,
Mohammad Lotfollahi, Hamid Alinejad-Rokny

February 15, 2024

## 1 Identifying spatial domain

#### 1.0.1 SpaCell

*Results:* SpaCell was tested on prostate cancer [1] and amyotrophic lateral sclerosis [2]. The results prove that the SpaCell outperformed all methods that used either gene expression or histology imaging by 8-14% improvement in cell-type clustering results (accuracy, precision, F-score, and AUC) and 4% in the classification results.

#### 1.0.2 stLearn

*Method:* The first step is the normalization of gene expression with spatial information, and H&E tissue images. Regarding spot $S_i$ in spatial data, there is a neighborhood spot $S_j$ if the distance between those centres $PD_{ij}$ is shorter than the radial of the predefined disk smoothing ($PD_{ij} < r$). After identifying neighbor's spots, the morphological similarity was measured by the corresponding HE images. StLearn then feeds images to the CNN model to extract numerical features from images with network weights pre-trained on the ImageNet dataset. The output from ResNet50 is a 2048-dimensional vector which was reduced to 50 by the principal component analysis (PCA) algorithm [3]. Therefore, the morphological distance ($MD$) can be calculated by the cosine distance between the obtained latent features $M_i$ and $M_j$ as:

$$MD(S_i, S_j) = \frac{M_i.M_j}{\|M_i\|.\|M_j\|} \tag{1}$$

The normalization of gene expression, called SME normalization, can be performed as:

$$GE'_i = GE_i + \frac{\sum_{j=1}^{n} GE_j.MD_{ij}}{n} \tag{2}$$

where $GE'_i$ is the normalized gene expression spot $S_i$. $GE_i$ and $GE_j$ are the raw gene expression for spot $S_i$ and its $n$ neighbor spots $S_j$.
*Results:* StLearn examined twelve human and mouse brain datasets and achieved a greater Adjusted Rand Index (ARI) value compared to the SpatialLIBD (a graph-based clustering method) [4] and detected two more tissue layers than Seurat.

#### 1.0.3 SpaGCN

*Method:* In the pre-processing step, SpaGCN eliminated each gene expression that appeared in less than three spots and stored the rest in a matrix along with the two spatial coordinates of each sample. SpaGCN normalized the given spot's gene count by dividing them by the total count across all genes, multiplied them by 10,000, and then transformed them into a natural log scale. SpaGCN then constructed an undirected graph $G(V, E)$ to identify the spatial domain, in which the edge weights were specified by distances between each spot $\in V$. Given the spatial gene expression $(x, y)$, SpaGCN added a new dimension $z$ by considering the pixel at coordinate $(x_{pv}, y_{pv})$ from

the spot $v$ in the histology image. First, it draws a square centred on $(x_{pv}, y_{pv})$ containing $50 \times 50$ pixels and then calculated the mean color value $(r_v, g_v, b_v)$, which $z_v$ can be measured as:

$$z_v = \frac{r_v \times V_r + g_v \times V_g + b_v \times V_b}{V_r + V_g + V_b} \tag{3}$$

where $V_{r,g,b} = Variance(r_v, g_v, b_v)$ for all $v \in V$. Second, SpaGCN rescaled $z_v$ as:

$$z_v^* = \frac{z_v - \mu_z}{\sigma_z} \times max(\sigma_x, \sigma_y) \times s \tag{4}$$

where $\mu_z$ represents the mean of $z_v$, and $\sigma_{x,y,z}$ are the standard deviations of $x_v$, $y_v$, and $z_v$, for all $v \in V$, and $s$ is a scaling factor. Thus, $(x_v, y_v, z_v^*)$ denotes the three dimensional space for each spot $v$ in a graph. To sum up, SpaGCN measured the weight of the edge between two spots $v_1$ and $v_2$ as:

$$w(v_1, v_2) = \exp(-\frac{d(v_1, v_2)^2}{2l^2}) \tag{5}$$

where $d(v_1, v_2)$ is the Euclidean distance between the two spots and $l$ is a hyper-parameter.
*Results:* The authors applied SpaGCN on five public datasets, four including sequencing-based data and one including MERFISH. SpaGCN recognized more spatial domains compared to other methods and obtained a higher ARI value than k-means and Louvain's [5] algorithm.

### 1.0.4 SEDR

*Method:* SEDR has three main steps: (1) learning latent features from the gene expression matrix $X$ and reconstructing it as $X'$ by deep autoencoder, (2) embedding the spatial information by using VGAEs, and (3) employing an unsupervised deep embedded clustering (DEC) to group the cells into different categories.
In the first step, SEDR obtains the latent features $Z_f$ from the output of the encoder part of the autoencoder (with two fully connected layers). Next, low-dimensional representation $Z_f$ and spatial embedding $Z_g$, obtained by step two, were concatenated into latent representation $Z$. SEDR trained the AE by maximizing the similarity between $X$ and $X'$ (the output of the decoder part) using the MSE loss function. In the second step, SEDR constructs the adjacency matrix $A$ by the ten nearest neighbors obtained from the Euclidean distances between image coordinates. In other words, SEDR embeds the spatial information and learns the graph embedding $Z_g$ via VGAE (parameterized by a two-layer GCN) from the adjacency matrix and its degree matrix $D$. While the reconstruction matrix $A'$ obtained by $Z_g.Z_g^T$, SEDR optimized the $Z_g$ by minimizing the cross-entropy between $A$ and $A'$ and KL divergence between p(Z—A) and its prior, simultaneously. In the last step, SEDR performs an unsupervised clustering method (DEC) [6] on the latent feature representation $Z$ to enhance the compactness of the learned features. SEDR then uses an SGD algorithm to optimize its parameters for clustering.
*Results:* The authors benchmarked SEDR against Seurat as a method that uses only gene expression, and Giotto, stLearn [7], SpaGCN [8], and BayesSpace [9] as methods which integrate gene expression and spatial information in their approaches. They tested these methods on the human dorsolateral prefrontal cortex (DLPFC) [4] and 10x Visium spatial transcriptomics data of human breast cancer and showed that the SEDR achieved higher ARI value than all other methods, even those with histology images (stLearn and SpaGCN). In addition to the high clustering performance of SEDR, the latent representation learned by SEDR can be effectively used in two procedures, i.e., (a) batch effect correction, in which SEDR could remove the batch effect in DLPFC data, and (b) tumour heterogeneity estimation in human breast cancer and high-resolution spatial data such as the mouse olfactory bulb dataset.

### 1.0.5 STAGATE.

*Method:* STAGATE constructs a spatial neighbor network (SNN) through two options. (a) Creating GAT network with adjacency matrix $A$ to convert the spatial information into the undirected graph according to the predefined radius $r$, where the matrix elements equal 1 if the Euclidean distance between two spots is less than $r$. The number of neighbors, and the parameter $r$ were defined based on the SRT datasets. (b) Cell type aware SNN obtained via pruning the GAT network relevant to pre-clustering gene expressions. These two modules can adaptively be selected as the input of the graph attention layer. STAGATE sets the encoder into the two neural network layers, where just the first layer was adopted to the attention layer. The two layers can be obtained as:

$$h_i^1 = \sum_{j \in S_i} att_{ij}^1 \sigma(W_1 h_j^0), (layer1) \tag{6}$$

$$h_i^2 = \sigma(W_2 h_i^1), (layer2) \tag{7}$$

where $h_i^1$ is the input gene expression spot $i$, $W$ is the trainable weight matrix, $S_i$ is the neighboring set of spot $i$, $\sigma$ is the non linear activation function, and $att_{ij}$ is the output of the graph attention layer measured as:

$$e_{ij}^1 = Sigmoid(v_s^{1^{(T)}} \sigma(W_1 h_i^0) + v_r^{1^{(T)}} \sigma(W_1 h_j^0)), \tag{8}$$

where $v_s^1$ and $v_r^1$ are the trainable vectors. The output of the attention layer can be measured by normalizing the $e_{ij}$ via the soft-max activation function:

$$att_{ij}^1 = \frac{\exp(e_{ij}^1)}{\sum_{i \in S_i} \exp(e_{ij}^1)}, \tag{9}$$

The $att_{ij}$ can be obtained through Graph attention convolution SNN ($att^{spatial}$) or cell type-aware SNN ($att^{aware}$). In other words, STAGATE multiplies the hyper-parameter $\alpha$ into the two obtained SNN in an adaptive way as follows:

$$att_{ij} = (1-\alpha)att_{ij}^{spatial} + \alpha att_{ij}^{aware}, \tag{10}$$

STAGATE sets the decoder part as the encoder, which receives the latent feature as an input and reverses it into the reconstructed gene expression input $h$. Same as the encoder, STAGATE uses the attention layer in the first layer of the decoder. Ultimately, STAGATE minimizes the $\sum_{i=1}^{N} \|x_i - h_i\|_2$ loss function and updates the trainable weights. Then, it performs mclust [10], and Louvain clustering algorithms for the labeled and unlabeled data on the learned features, respectively.
*Results:* The authors applied STAGATE to the DLPFC dataset (almost 4789 spots), Stereo-seq mouse olfactory bulb data (19109 spots), and Slide-seqV2 mouse hippocampus data (19285 spots), and mouse olfactory bulb data (20139 spots) obtained by different SRT technologies. STAGATE performed better in clustering than spatial methods, including SEDR, StLearn, BayesSpace (a Bayesian model), and the non-spatial method SCANPY [11] (a Python-based implementation framework). It was also able to accurately reduce noise in the DLPFC dataset and enhance the gene expression profiling ability within spatial domains. Removing batch effects in the seven hippocampus sections profiled by Slide-seq was another ability of STAGATE, which enables extracting 3D patterns by utilizing 3D SNN.

#### 1.0.6 RESEPT

*Results:* The authors selected various benchmarking metrics including ARI, rand index (RI), and Fowlkes–mallows index (FM) to evaluate the obtained segmentation and manual annotation. The Moran's I and PSNR (see Supplementary Table S1) metrics were used to assess the quality of the predicted segmentation map and 3D embedding evaluation, respectively. They applied RESEPT on 16 SRT samples (12 published and four in-house including two Alzheimer disease (AD), health, and tumour samples) from the human brain cortex region and compared RESEPT to SpaGCN, stLearn, and other ML methods. They achieved a higher ARI value than other models. Moreover, RGB images generated from the RNA velocity can reveal clear domains in AD samples (higher Moran's I compared to the models that use gene expression as an input). Given this performance, they analyzed the glioblastoma dataset published by 10x Genomics to evaluate the clinical applications of RESEPT in the oncology field and found RESEPT accurately identified the eight segmented areas. Those areas represented a good understanding of glioblastoma heterogeneity. Furthermore, providing two input options for the graph autoencoder is the novel quality of RESEPT, allowing to investigate well-differentiated architectures in the SRT data.

#### 1.0.7 ECNN

*Results:* The authors used the SRT dataset in [12] including seven H&E-stained tissue slides and applied the Calinski–Harabasz [13] method to detect the number of clusters per slide. First, they obtained the Dice index matrix to compare predicted clusters to manual annotations, which ECNN achieved coherent regions even using other clustering methods, such as K-means, Gaussian mixture model (GMM) [14], and spectral clustering. The results also illustrate that the automatically obtained color maps successfully separated different heterogeneity regions. It was shown that the pre-trained ECNN using ImageNet could considerably downgrade the performance.

#### 1.0.8 JSTA

*Results:* The performance of JSTA was tested first on the synthetic data from mouse hippocampus generated based on the NCTT [15], in which JSTA outperformed both watershed and pciSeq [16] algorithms in cell type identification even with a small number of genes. JSTA was also tested on other MERFISH and scRNAseq datasets. The obtained results demonstrated that JSTA can segment cells in MERFISH data, which are highly correlated with their scRNAseq counterparts. Also, the previous test was repeated with another MERFISH dataset [17] including 134 genes, confirming the efficiency of JSTA. Finally, JSTA was evaluated on an osmFISH dataset from the mouse somatosensory cortex with the 35 genes [18], which JSTA successfully mapped 142 high-resolution cell types in this region.

#### 1.0.9 conST

*Results:* The datasets used were mouse hypothalamus MERFISH, and mouse visual cortex seqFISH for image-based methods; and two datasets generated by 10x genomics, and mouse olfactory bulb Stereo-seq for sequencing-based methods. The Leiden algorithm was used to cluster the spatial domain with the obtained embeddings.

conST achieved an ARI of 0.65, approximately a 10% increase compared to other state-of-the-art methods in the sequencing-based datasets, and demonstrated high performance on the image-based datasets. Since there is no ground truth for the MERFISH dataset, the performance of the clustering method was compared with three other unsupervised metrics SC, CHS, and DBI (see Supplementary Table S1).

## 1.1 Identifying spatially variable genes

### 1.1.1 CoSTA

*Method:* The proposed method consists of two steps: clustering and neural network training. In the first step, CoSTA passes the normalized images through the CNN network (ConvNet), which consists of three convolution layers. Each layer is followed by a batch normalization layer and max-pooling layer. The flattened layer after the last max-pooling layer is called as the spatial features of the gene expression data. To cluster features, the method applies the L2-normalization and UMAP (an unsupervised dimension reduction method) to the feature vector, respectively. Then, it performs UMAP to reduce the dimensions to 30 features and cluster all samples by GMM. The authors tested various cluster numbers in Slide-seq data and proved that the proposed network could converge regardless of cluster number during training, but 30 clusters showed better specificity. The purpose of clustering is to generate labels for training ConvNet. Moreover, each gene is assigned to a cluster by an auxiliary target distribution, which is the likelihood of the gene belonging to the cluster. The probability of the soft assignment of each sample $i$ can be measured based on the Euclidean distances $d_i$ to cluster centroids ci as follows:

$$p(y = i \mid x) = \frac{e^{1/d_i}}{\sum_{i=1}^{N} e^{1/d_i}} \tag{11}$$

where, $N$ is the total number of clusters. The auxiliary target probability of $jth$ sample belonging to the $ith$ cluster $q_{ij}$ is calculated using Eq 12.

$$q_{ij} = \frac{p_{ij}^2/f_i}{\sum_{i=1}^{N} p_{ij}^2/f_i} \tag{12}$$

where, $f_i = \sum_{j=1}^{M} p_{ij}$, and $p_{ij}$ is obtained through Eq 11.
Once the label generation in the first step is finished, the second step adds a fully connected layer (FC), with the softmax activation function, to the last max-pooling layer. The output size of the FC layer is equal to the number of clusters in the previous step and produces the probability of the input gene belonging to each cluster. The method uses the FC layer just during training, and it would be discarded in the first mentioned step. Moreover, it retains trained ConvNet just for feature extraction in the other stages.
*Result:* The CoSTA model was tested on three different types of datasets. a. Typical image datasets, such as MNIST, USPS-digit, and Fashion, to assess the network ability of spatial pattern recognition and correlation in the absence of overlapping. b. Simulation datasets, consist of five synthetic datasets with different noise levels, to determine the robustness of the proposed method against varying degrees of noise: c. MERFISH and Slide-seq dataset. In the first category, the proposed method was benchmarked against supervised and unsupervised learning methods and achieved less accuracy than supervised methods in three datasets. With regards to the NMI metric (see Supplementary Table S1), CoSTA obtained higher NMIs than other cluster learning methods when it was applied to MNIST and Fashion and was scored as the second-best model in USPS-digit dataset. To confirm that the network learns features based on pixel correlation rather than pixel-wise, CoSTA was tested on the shuffled pixel positions. The authors found that CoSTA could not distinguish the spatial patterns in the synthetic datasets, and it proved that the CoSTA learns features based on the correlations between neighboring pixels. CoSTA recognized the quantitative similarity between genes in the Merish dataset, which achieved less sensitivity and more specificity (identified 133 spatially expressed (SE) genes) than the Spark (145 SE genes) and SpatialDE (139 SE genes) methods. The CoSTA results on Slide-seq data demonstrated that it identified spatial patterns-dependent, accurately. They also repeated the shuffling approach on Slide-seq data, which suggested again that the learned patterns by the proposed method are highly related to the spatial expression pattern, even using actual biological data.

### 1.1.2 ST-Net

*Results:* ST-Net was trained on a breast cancer spatial transcriptomics dataset, including 30,612 spots in 68 breast tissue sections from 23 patients using leave-one-patient-out cross-validation, where it was repetitively trained on 22 patients and tested on the remaining held-out patient. ST-Net achieved a mean square error of 0.31 and acceptable Pearson's correlation (the average of 0.33 across all 234 genes), in which 102 of the 250 genes were correlated positively in almost 20 patients. Moreover, ST-Net was externally validated on the 10x Genomics breast cancer dataset and the breast cancer samples of the Cancer Genome Atlas (TCGA). ST-Net predicted 207 of the 234 genes and 177 of 249 genes with a positive correlation and 0.73 and 0.83 area under curve (AUC) in the former

and latter datasets, respectively. All results were obtained without any prior modifications, proving the robustness and generalisability of the proposed deep model in the breast cancer dataset. The authors plotted UMAP for visualizing the latent feature vector and found that the latent feature can separate the tumour and non-tumour spots, which can be leveraged in clustering and cell-type composition.

### 1.1.3 HisToGene

*Results:* The results consisted of three parts: a. Applying on the HER2 + breast cancer dataset [21] including 36 tissue sections from 8 patients; b. Generalising the model on the cutaneous squamous cell carcinoma (cSCC) dataset [22], including 12 tissue sections from 4 patients; c. Clustering spatial domains using predicted gene expressions on the HER2 + breast cancer dataset. In the first part, the authors trained the model on 32 sections from 7 patients with leave-one-out (32-fold) cross-validation, in which they trained the model using 31 tissue and tested on the one remaining tissue. They compared the HisToGene with ST-Net, which consistently outperformed ST-Net in correlation. In the second part, despite the better performance of the proposed method rather than ST-Net, neither model could achieve acceptable (high accuracy) results due to the low resolution of spots. The authors performed K-means clustering in the third part, in which HisToGene obtained a higher ARI than ST-Net.

### 1.1.4 CNNTL

*Result:* The CNNTL approach was tested on the Cortex study dataset obtained from 42 donors. The metric is rank-1 accuracy at the level of images, which is the proportion of images for which the Euclidean distance of their embeddings computes the closest image of the same gene. The CNNTL achieved rank-1 accuracy of 38.3%, which performed better than single ResNet or random models. Also, with the learned embeddings by CNNTL, the proposed method could successfully predict tissue source (AUC = 0.902), expression intensity (AUC = 0.898), laminar patterns (AUC = 0.879), and cell-type specificity (AUC = 0.805), which are higher than the results obtained from the baseline ResNet50. The authors validated the triplet model on the Schizophrenia study dataset (rank-1 score of 60.2%), and with the help of learned features, CNNTL achieved an AUC of 0.59 in predicting Schizophrenia.

### 1.1.5 DeepSpaCE

The DeepSpaCE was tested on a dataset from human breast cancer consisting of three tissue sections A, B, C, and related consecutive sections (D1–D3). First, the model was trained on the D2 for predicting three breast cancer-marker genes (SmoothL1 loss function), in which the model obtained 0.588 correlation coefficients between the measured and predicted values. Next, the authors used DeepSpaCE to predict cell-type clusters (Cross-Entropy Loss function) and utilized the obtained clusters via the Space Ranger software as a ground truth, which the proposed method achieved high recall value.

Supplementary Table 1: Evaluation metrics used in surveyed DL algorithms.

| **Evaluation method** | **Equations** | **Explanation** | **Model** |
|---|---|---|---|
| Entropy of mixing[23] | $E = \sum_{i=1}^{c} x_i log(x_i)$ | This metric quantifies the extent of the intermingling of cells from different batches. $x_i$ is the proportion of cell $i$ and $c$ is the number of batches. | gimVI |
| Kullback–Leibler (KL) divergence [24] | $D_{KL}(P \parallel Q) = \sum_{x \in X} P(x) log(\frac{P(x)}{Q(X)})$ | The Kullback–Leibler divergence measures how the two distributions $P$ and $Q$ on space $X$ are different. | gimVI |
| Jaccard Index | $J(X,Y) = \frac{\|X \cap Y\|}{\|X \cup Y\|}$ | JI metric measures the similarity between two sets $X$ and $Y$. | gimVI |
| NMI[25] | $NMI = \frac{2I(A,B)}{H(A)+H(B)}$ | The Normalized Mutual Information (NMI) is a cluster comparison metric between the two clusters, A and B. H and I are the entropy and joint entropy of the two clusters, respectively. The output are bounded in [0,1], where 1 means the two clusters are identical. | CoSTA<br>stPlus |
| Adjusted Rand index[26] | $ARI = \frac{RI - expectedRI}{maxRI - expectedRI}$<br>$RI = \frac{TP+TN}{TP+FP+FN+TN}$ | The Adjusted Rand index measures the similarity between two clusters, where the RI stands for the rand index. | stlearn<br>SpaGCN<br>SEDR<br>STAGATE<br>HisToGene<br>stPlus<br>RESEPT<br>conST |
| Moran's I[19] | $I = \frac{N}{W} \frac{\sum_i \sum_j [w_{ij}(x_i - \hat{x})(x_j - \hat{x})]}{\sum_i (x_i - \hat{x})^2}$ | The Moran's I is a metric of spatial autocorrelation, and the range is between [-1,1], where values closer to 1 indicate a better spatial pattern. Where the $x_i$ and $x_j$ are the gene expression of the spot $i$ and $j$, $w_{ij}$ is the spatial distance between two spots, $\hat{x}$ is the mean expression of gene, $W$ is the sum of $w_{ij}$. | SpaGCN<br>SEDR<br>STAGATE<br>RESEPT<br>conST |
| Pearson's correlation[27] | $\rho_{X,Y} = \frac{\sum(X_i - \hat{X})(Y_i - \hat{Y})}{\sqrt{\sum(X_i - \hat{X})^2}\sqrt{\sum(Y_i - \hat{Y})^2}}$ | The Pearson's correlation measures the similarity between two objects which produce the score from -1 and 1, representing 1 (high correlation), 0 (uncorrelated), and -1 (inverse correlation). | ST-Net<br>HisToGene<br>JSTA<br>DSTG |

Supplementary Table 1: Evaluation metrics used in surveyed DL algorithms.

| **Evaluation method** | **Equations** | **Explanation** | **Model** |
|---|---|---|---|
| Spearman Correlation[28] | $\rho_s = 1 - \frac{6\sum(r_X - r_y)^2}{n(n^2-1)}$ | The Spearman Correlation measures the degree of association between two variables $X$ and $Y$, where $r_X$ and $r_Y$ are the ranks of the two variables. | gimVI<br>stPlus |
| Root mean squared error[29] | RMSE=$\sqrt{\frac{1}{n}\sum_{i=1}^{n} e_i^2}$ | The RMSE is the static metric for evaluating the model, where $e_i$ denotes the error between the predicted and actual value of sample $i$, and $n$ is the number of total samples. | ST-Net<br>XFuse |
| AUC | - | The ROC curve can be plotted based on the true-positive rate(y-axis) and false-positive rate(x-axis), which show the trade-off between them. The area under the obtained curve (AUROC or AUC) can illustrate the ML model's performance. | SpaCell<br>ST-Net<br>GCNG |
| AMI[30] | $AMI = \frac{I(A,B) - E(I(A,B))}{avg[H(A),H(B)] - E[I(A,B)]}$ | The Adjusted mutual information is the clustering metric to assess the similarity between the two clusters A and B. $E(.)$ denote the expectation function. (Refer to NMI formula for the other functions). | stPlus<br>RESEPT |
| Homo[31] | $1 - \frac{I(T\|P)}{I(T)}$ | The homogeneity score estimate whether the predicted clusters $P$ contain only object of the same population. The output equals 1, if all the objects through the same cluster correspond to the same population. | stPlus |
| Dice index[32] | $D(G,C) = \frac{2\|G \cap C\|}{\|G\| + \|C\|}$ | The Dice index numerically assesses the shared region between ground-truth $G$ and the obtained cluster $c$. The output is between [0,1], which 1 denotes high similarity. | ECNN |
| RMI[32] | $R_{F,C} = \frac{I(F(In)) - I(F(Out))}{I(F_i(In)) + I(F_i(Out))}$ | The relative mean intensity value measures whether the obtained cluster $C$ is relevant to the gene expression in ST data. First, thousand gene expressions are summarized to the 25 gene factors $F_i$ by the methods in [1, 12], then the mean factor intensity $I$ inside and outside of the cluster C are measured. | ECNN |

Supplementary Table 1: Evaluation metrics used in surveyed DL algorithms.

| Evaluation method | Equations | Explanation | Model |
|---|---|---|---|
| MAE | $MAE = \frac{\sum_{i=1}^{n} \lvert y_i - x_i \rvert}{n}$ | The mean absolute error calculates the average absolute value of the difference between each predicted object $x_i$ and the ground truth $y_i$ for all $n$ samples. | GIST |
| FM[33] | $FM = \sqrt{precision.recall}$ | The Fowlkes–Mallows index measures the consistency of obtained results between the obtained cluster and ground truth. The results are $\in [0,1]$, which 1 denoting perfect clustering. | RESEPT |
| PSNR[33] | $PSNR = \frac{\sum_{i=1}^{p} 10log10(\frac{MAX_i^2}{MSE_i} \times a_i)}{\sum_{i=1}^{p} a_i}$ | The peak signal-to-noise ratio metric assess the similarity between the color distribution of an RGB image and its corresponding labeled segmentation map, where $a_i$ is the number of pixels in $i^{th}$ segment, $MAX_i$ represents the maximum pixel value in $i^{th}$ segment, $MSE_i$ denote as pixel-wise mean square error of the $i^{th}$ segment, and $p$ is the total number of segmented areas. The higher value of PSNR implies the better quality of mapped RGB images. | RESEPT |
| JSD[34] | $JSD(P^i \vert\vert Q^i) = \frac{1}{2}\sum_{k\in[1,...,C]} p_i^k log(\frac{p_i^k}{(p_i^k+q_i^k)/2}) + \frac{1}{2}\sum_{k\in[1,...,C]} q_i^k log(\frac{q_i^k}{(p_i^k+q_i^k)/2})$ | The Jensen–Shannon divergence (JSD) score is a similarity measurement, which obtain the similarity between the probability distribution of ground truth composition $P^i = (p_1^i, p-2^i, ..., p_C^i)$ and distribution of predicted composition $Q^i = (q_1^i, q-2^i, ..., q_C^i)$ at spot $i$. The lower JSD score exhibits a higher similarity. | DSTG |
| Silhouette Coefcient (SC)[26] | $SC = \frac{b-a}{max(a,b)}$ | This metric evaluates the performance of unsupervised clustering, where $b$ represents the average nearest cluster distance for every sample and $a$ stands for the mean cluster centroid distance. The result would range from [-1,1], where a higher value indicates a better clustering performance. | conST |

Supplementary Table 1: Evaluation metrics used in surveyed DL algorithms.

| **Evaluation method** | **Equations** | **Explanation** | **Model** |
|---|---|---|---|
| Calinski Harabasz Score (CHS)[35] | $CHS = \frac{trB_k}{trW_k} \times \frac{n_E - k}{k-1}$ | Also known as the Variance Ratio Criterion, which measures the clustering performance when no ground truth is available. $tr$ is a trace between-group dispersion matrix $B_k$ and within-cluster dispersion matrix $W_k$, where $n_E$ is the data size and $k$ represents the cluster's number. A higher score represents a better clustering performance. | conST |
| Davies Bouldin Index(DBI)[35] | $\frac{1}{k} \sum_{i=1}^{k} max(\frac{s_i + s_j}{d_{ij}})$ | This metric measures the average the similarity between each cluster $i = 1, ..., k$ and most similar one $j$, where $s_i$ and $s_j$ are cluster diameter and $d_{ij}$ denotes the distance between cluster centroids $i$ and $j$. A lower DBI stands for a model with better separation between the clusters. | conST |
| Recall | $Recall = \frac{TP}{TP+FN}$ | It measures the ratio of positive class (TP) out of all positive examples.FN: false negative | DeepSpaCE |

Supplementary Table 2: SRT data sources used by different DL algorithms.

| Title | Number of genes/spots | Sources |
|---|---|---|
| zebrafish embryo | 47 genes | https://dropbox.com/s/ev78jelev0jgu5s/seurat_files_zfin.zip?dl=1 |
| Drosophila embryo | 84 genes | - |
| mouse frontal cortex | 32,285 genes | https://support.10xgenomics.com/spatial-gene-expression/datasets/1.1.0/V1_Mouse_Brain_Sagittal_Anterior |
| mSMS [18] | 33 genes | - |
| mPFC [36] | 166 genes | - |
| mouse hypothalamic MERFISH [17] | 134 genes | https://figshare.com/articles/dataset/Raw_images/14531553 |
| mouse visual cortex seqFISH [37] | 25 genes | https://spatial.rc.fas.harvard.edu |
| DLPFC (12 slices) [4] | 33,538 genes (slice 151673) | http://spatial.libd.org/spatialLIBD |
| mouse olfactory bulb Stereo-seq [38] | 19,109 spots | https://github.com/JinmiaoChenLab/SEDR_analyses |
| Slide-seq [39] | - | https://portals.broadinstitute.org/single_cell/study/slide-seq-study |
| Slide-seqV2 mouse hippocampus | 19,285 spots | https://singlecell.broadinstitute.org/single_cell/study/SCP815 |
| Slide-seqV2 mouse olfactory bulb | 20,139 spots | https://singlecell.broadinstitute.org/single_cell/study/SCP815 |
| MERFISH MOp | 254 genes | https://doi.brainimagelibrary.org/doi/10.35077/g.21 |
| STARmap [36] | 1020 genes | - |
| human brain datasets on 10x Visium | - | https://www.10xgenomics.com/products/spatial-gene-expression |
| HDST | - | https://singlecell.broadinstitute.org/single_cell |
| Globus | - | http://research.libd.org/globus/ |
| human breast cancer | - | https://www.10xgenomics.com/resources/datasets/ |
| seqFISH+ mouse cortex [40] | 10,000 genes(913 cells) | https://github.com/CaiGroup/seqFISH-PLUS |
| seqFISH+ mouse olfactory bulb | [40],10,000 genes (2050 cells) | https://github.com/CaiGroup/seqFISH-PLUS |
| MERFISH [41] | 10,050 genes | https://www.pnas.org/content/116/39/19490/tab-figuresdata |

Supplementary Table 2: SRT data sources used by different DL algorithms.

| Title | Number of genes/spots | Sources |
|---|---|---|
| breast-cancer (68 tissues) | 30,612 genes | http:/www.spatialtranscriptomicsresearch.org |
| 10x Spatial Genomics (breast cancer) | - | https://wp.10xgenomics.com/spatial-transcriptomics |
| mouse olfactory bulb (MOB)[42] | 16,218 genes (262 spots) | https://drive.google.com/drive/folders1C4l3lBaYl7uuV2AA2o0WDzO_mkc_b0pv?usp=sharing |
| mouse posterior cerebrum | 31,053 genes (3,353 spots) | https://support.10xgenomics.com/spatial-gene-644expression/datasets/1.0.0/V1_Mouse_Brain_Sagittal_Posterior |
| human primary pancreatic cancer [43] | 16,448 genes (224 spots) | - |
| MERFISH mouse hypothalamus [17] | 161 genes (5,665 cells) | https://datadryad.org/stash/dataset/doi:10.5061/dryad.8t8s248 |
| osmFISH [44] | 35 genes | - |
| HER2+ breast cancer [21] | 36 tissue sections (180 spots per section) | https://github.com/almaan/her2st |
| cutaneous squamous cell carcinoma (cSCC) [22] | 12 tissue sections | - |
| Prostate Cancer ST [12] | 23,282 spots | - |
| prostate cancer [1] | 12,000 genes (12 tissue slides) | - |
| amyotrophic lateral sclerosis [2] | - | - |
| Cortex Study [45] | 1,000 genes (human cerebral cortex) | https://figshare.com/s/43ebba2711adc3ccdc13 |
| Schizophrenia Study [46] | 78 genes | https://figshare.com/s/43ebba2711adc3ccdc13 |

## References


[1] E. Berglund, J. Maaskola, N. Schultz, S. Friedrich, M. Marklund, J. Bergenstråhle, F. Tarish, A. Tanoglidi, S. Vickovic, L. Larsson, et al. Spatial maps of prostate cancer transcriptomes reveal an unexplored landscape of heterogeneity. *Nature communications*, 9(1):1–13, 2018.

[2] S. Maniatis, T. Äijö, S. Vickovic, C. Braine, K. Kang, A. Mollbrink, D. Fagegaltier, Ž. Andrusivová, S. Saarenpää, G. Saiz-Castro, et al. Spatiotemporal dynamics of molecular pathology in amyotrophic lateral sclerosis. *Science*, 364(6435):89–93, 2019.

[3] H. Abdi and L. J. Williams. Principal component analysis. *Wiley interdisciplinary reviews: computational statistics*, 2(4):433–459, 2010.

[4] K. R. Maynard, L. Collado-Torres, L. M. Weber, C. Uytingco, B. K. Barry, S. R. Williams, J. L. Catallini, M. N. Tran, Z. Besich, M. Tippani, et al. Transcriptome-scale spatial gene expression in the human dorsolateral prefrontal cortex. *Nature neuroscience*, 24(3):425–436, 2021.

[5] V. D. Blondel, J.-L. Guillaume, R. Lambiotte, and E. Lefebvre. Fast unfolding of communities in large networks. *Journal of statistical mechanics: theory and experiment*, 2008(10):P10008, 2008.

[6] J. Xie, R. Girshick, and A. Farhadi. Unsupervised deep embedding for clustering analysis. In *International conference on machine learning*, pages 478–487. PMLR, 2016.

[7] D. Pham, X. Tan, J. Xu, L. F. Grice, P. Y. Lam, A. Raghubar, J. Vukovic, M. J. Ruitenberg, and Q. Nguyen. stlearn: integrating spatial location, tissue morphology and gene expression to find cell types, cell-cell interactions and spatial trajectories within undissociated tissues. *bioRxiv*, 2020.

[8] J. Hu, X. Li, K. Coleman, A. Schroeder, N. Ma, D. J. Irwin, E. B. Lee, R. T. Shinohara, and M. Li. Spagcn: Integrating gene expression, spatial location and histology to identify spatial domains and spatially variable genes by graph convolutional network. *Nature methods*, 18(11):1342–1351, 2021.

[9] E. Zhao, M. R. Stone, X. Ren, T. Pulliam, P. Nghiem, J. H. Bielas, and R. Gottardo. Bayesspace enables the robust characterization of spatial gene expression architecture in tissue sections at increased resolution. *bioRxiv*, 2020.

[10] C. Fraley, A. Raftery, and L. Scrucca. mclust: Normal mixture modeling for model-based clustering, classification, and density estimation. *R package version*, 4(7), 2014.

[11] F. A. Wolf, P. Angerer, and F. J. Theis. Scanpy: large-scale single-cell gene expression data analysis. *Genome biology*, 19(1):1–5, 2018.

[12] A. Erickson, E. Berglund, M. He, M. Marklund, R. Mirzazadeh, N. Schultz, L. Bergenstråhle, L. Kvastad, A. Andersson, J. Bergenstråhle, et al. The spatial landscape of clonal somatic mutations in benign and malignant tissue. *bioRxiv*, 2021.

[13] T. Caliński and J. Harabasz. A dendrite method for cluster analysis. *Communications in Statistics-theory and Methods*, 3(1):1–27, 1974.

[14] C. Rasmussen. The infinite gaussian mixture model. *Advances in neural information processing systems*, 12, 1999.

[15] E. S. Lein, M. J. Hawrylycz, N. Ao, M. Ayres, A. Bensinger, A. Bernard, A. F. Boe, M. S. Boguski, K. S. Brockway, E. J. Byrnes, et al. Genome-wide atlas of gene expression in the adult mouse brain. *Nature*, 445(7124):168–176, 2007.

[16] X. Qian, K. D. Harris, T. Hauling, D. Nicoloutsopoulos, A. B. Muñoz-Manchado, N. Skene, J. Hjerling-Leffler, and M. Nilsson. Probabilistic cell typing enables fine mapping of closely related cell types in situ. *Nature methods*, 17(1):101–106, 2020.

[17] J. R. Moffitt, D. Bambah-Mukku, S. W. Eichhorn, E. Vaughn, K. Shekhar, J. D. Perez, N. D. Rubinstein, J. Hao, A. Regev, C. Dulac, et al. Molecular, spatial, and functional single-cell profiling of the hypothalamic preoptic region. *Science*, 362(6416):eaau5324, 2018.

[18] S. Codeluppi, L. E. Borm, A. Zeisel, G. La Manno, J. A. van Lunteren, C. I. Svensson, and S. Linnarsson. Spatial organization of the somatosensory cortex revealed by osmfish. *Nature methods*, 15(11):932–935, 2018.

[19] H. Li, C. A. Calder, and N. Cressie. Beyond moran's i: testing for spatial dependence based on the spatial autoregressive model. *Geographical Analysis*, 39(4):357–375, 2007.

[20] S. Sun, J. Zhu, and X. Zhou. Statistical analysis of spatial expression patterns for spatially resolved transcriptomic studies. *Nature methods*, 17(2):193–200, 2020.

[21] A. Andersson, L. Larsson, L. Stenbeck, F. Salmén, A. Ehinger, S. Z. Wu, G. Al-Eryani, D. Roden, A. Swarbrick, Å. Borg, et al. Spatial deconvolution of her2-positive breast cancer delineates tumor-associated cell type interactions. *Nature communications*, 12(1):1–14, 2021.

[22] A. L. Ji, A. J. Rubin, K. Thrane, S. Jiang, D. L. Reynolds, R. M. Meyers, M. G. Guo, B. M. George, A. Mollbrink, J. Bergenstråhle, et al. Multimodal analysis of composition and spatial architecture in human squamous cell carcinoma. *Cell*, 182(2):497–514, 2020.

[23] L. Haghverdi, A. T. Lun, M. D. Morgan, and J. C. Marioni. Batch effects in single-cell rna-sequencing data are corrected by matching mutual nearest neighbors. *Nature biotechnology*, 36(5):421–427, 2018.

[24] D. J. MacKay, D. J. Mac Kay, et al. *Information theory, inference and learning algorithms*. Cambridge university press, 2003.

[25] N. X. Vinh, J. Epps, and J. Bailey. Information theoretic measures for clusterings comparison: Variants, properties, normalization and correction for chance. *Journal of Machine Learning Research*, 11(95):2837–2854, 2010.

[26] R. Sinnott, H. Duan, and Y. Sun. Chapter 15 - a case study in big data analytics: Exploring twitter sentiment analysis and the weather. In R. Buyya, R. N. Calheiros, and A. V. Dastjerdi, editors, *Big Data*, pages 357–388. Morgan Kaufmann, 2016.

[27] J. J. Berman. *Data simplification: taming information with open source tools*. Morgan Kaufmann, 2016.

[28] M. Meloun and J. Militký. 7 - correlation. In M. Meloun and J. Militký, editors, *Statistical Data Analysis*, pages 631–666. Woodhead Publishing India, 2011.

[29] T. Chai and R. R. Draxler. Root mean square error (rmse) or mean absolute error (mae). *Geoscientific Model Development Discussions*, 7:1525–1534, 2014.

[30] S. Romano, N. X. Vinh, J. Bailey, and K. Verspoor. Adjusting for chance clustering comparison measures. *The Journal of Machine Learning Research*, 17(1):4635–4666, 2016.

[31] J. Pauwels, A. Lamberty, and H. Schimmel. Homogeneity testing of reference materials. *Accreditation and quality assurance*, 3(2):51–55, 1998.

[32] E. Chelebian, C. Avenel, K. Kartasalo, M. Marklund, A. Tanoglidi, T. Mirtti, R. Colling, A. Erickson, A. D. Lamb, J. Lundeberg, et al. Morphological features extracted by ai associated with spatial transcriptomics in prostate cancer. *Cancers*, 13(19):4837, 2021.

[33] Y. Chang, F. He, J. Wang, S. Chen, J. Li, J. Liu, Y. Yu, L. Su, A. Ma, C. Allen, et al. Define and visualize pathological architectures of human tissues from spatially resolved transcriptomics using deep learning. *bioRxiv*, 2021.

[34] Q. Song and J. Su. DSTG: deconvoluting spatial transcriptomics data through graph-based artificial intelligence. *Briefings in Bioinformatics*, 22(5), 01 2021.

[35] Y. Liu, Z. Li, H. Xiong, X. Gao, and J. Wu. Understanding of internal clustering validation measures. In *2010 IEEE international conference on data mining*, pages 911–916. IEEE, 2010.

[36] X. Wang, W. E. Allen, M. A. Wright, E. L. Sylwestrak, N. Samusik, S. Vesuna, K. Evans, C. Liu, C. Ramakrishnan, J. Liu, et al. Three-dimensional intact-tissue sequencing of single-cell transcriptional states. *Science*, 361(6400):eaat5691, 2018.

[37] S. Shah, E. Lubeck, W. Zhou, and L. Cai. In situ transcription profiling of single cells reveals spatial organization of cells in the mouse hippocampus. *Neuron*, 92(2):342–357, 2016.

[38] A. Chen, S. Liao, M. Cheng, K. Ma, L. Wu, Y. Lai, J. Yang, W. Li, J. Xu, S. Hao, et al. Large field of view-spatially resolved transcriptomics at nanoscale resolution. *bioRxiv*, 2021.

[39] S. G. Rodriques, R. R. Stickels, A. Goeva, C. A. Martin, E. Murray, C. R. Vanderburg, J. Welch, L. M. Chen, F. Chen, and E. Z. Macosko. Slide-seq: A scalable technology for measuring genome-wide expression at high spatial resolution. *Science*, 363(6434):1463–1467, 2019.

[40] C.-H. L. Eng, M. Lawson, Q. Zhu, R. Dries, N. Koulena, Y. Takei, J. Yun, C. Cronin, C. Karp, G.-C. Yuan, et al. Transcriptome-scale super-resolved imaging in tissues by rna seqfish+. *Nature*, 568(7751):235–239, 2019.

[41] C. Xia, J. Fan, G. Emanuel, J. Hao, and X. Zhuang. Spatial transcriptome profiling by merfish reveals subcellular rna compartmentalization and cell cycle-dependent gene expression. *Proceedings of the National Academy of Sciences*, 116(39):19490–19499, 2019.

[42] P. L. Ståhl, F. Salmén, S. Vickovic, A. Lundmark, J. F. Navarro, J. Magnusson, S. Giacomello, M. Asp, J. O. Westholm, M. Huss, et al. Visualization and analysis of gene expression in tissue sections by spatial transcriptomics. *Science*, 353(6294):78–82, 2016.

[43] R. Moncada, D. Barkley, F. Wagner, M. Chiodin, J. C. Devlin, M. Baron, C. H. Hajdu, D. M. Simeone, and I. Yanai. Integrating microarray-based spatial transcriptomics and single-cell rna-seq reveals tissue architecture in pancreatic ductal adenocarcinomas. *Nature biotechnology*, 38(3):333–342, 2020.

[44] S. Codeluppi, L. E. Borm, A. Zeisel, G. La Manno, J. A. van Lunteren, C. I. Svensson, and S. Linnarsson. Spatial organization of the somatosensory cortex revealed by osmfish. *Nature methods*, 15(11):932–935, 2018.

[45] H. Zeng, E. H. Shen, J. G. Hohmann, S. W. Oh, A. Bernard, J. J. Royall, K. J. Glattfelder, S. M. Sunkin, J. A. Morris, A. L. Guillozet-Bongaarts, et al. Large-scale cellular-resolution gene profiling in human neocortex reveals species-specific molecular signatures. *Cell*, 149(2):483–496, 2012.

[46] A. Guillozet-Bongaarts, T. Hyde, R. Dalley, M. Hawrylycz, A. Henry, P. Hof, J. Hohmann, A. Jones, C. Kuan, J. Royall, et al. Altered gene expression in the dorsolateral prefrontal cortex of individuals with schizophrenia. *Molecular psychiatry*, 19(4):478–485, 2014.
